# In Silico Strategy for Targeting the mTOR Kinase at Rapamycin Binding Site by Small Molecules

**DOI:** 10.3390/molecules26041103

**Published:** 2021-02-19

**Authors:** Serena Vittorio, Rosaria Gitto, Ilenia Adornato, Emilio Russo, Laura De Luca

**Affiliations:** 1 Department of Chemical, Biological, Pharmaceutical and Environmental Sciences, University of Messina, Viale Palatucci 13, I-98168 Messina, Italy; svittorio@unime.it (S.V.); rgitto@unime.it (R.G.); ilenia_adornato@hotmail.it (I.A.); 2 Science of Health Department, School of Medicine, University “Magna Graecia” of Catanzaro, Viale Europa e Germaneto, I-88100 Catanzaro, Italy; erusso@unicz.it

**Keywords:** mTOR, molecular dynamics simulation, ternary complex FKBP12-rapamycin-FRB, molecular docking and structure-based virtual screening, pharmacophore modeling

## Abstract

Computer aided drug-design methods proved to be powerful tools for the identification of new therapeutic agents. We employed a structure-based workflow to identify new inhibitors targeting mTOR kinase at rapamycin binding site. By combining molecular dynamics (MD) simulation and pharmacophore modelling, a simplified structure-based pharmacophore hypothesis was built starting from the FKBP12-rapamycin-FRB ternary complex retrieved from RCSB Protein Data Bank (PDB code 1FAP). Then, the obtained model was used as filter to screen the ZINC biogenic compounds library, containing molecules derived from natural sources or natural-inspired compounds. The resulting hits were clustered according to their similarity; moreover, compounds showing the highest pharmacophore fit-score were chosen from each cluster. The selected molecules were subjected to docking studies to clarify their putative binding mode. The binding free energy of the obtained complexes was calculated by MM/GBSA method and the hits characterized by the lowest ΔG_bind_ values were identified as potential mTOR inhibitors. Furthermore, the stability of the resulting complexes was studied by means of MD simulation which revealed that the selected compounds were able to form a stable ternary complex with FKBP12 and FRB domain, thus underlining their potential ability to inhibit mTOR with a rapamycin-like mechanism.

## 1. Introduction

The mammalian target of rapamycin (mTOR) protein is a highly conserved eukaryotic Serine/Threonine (Ser/Thr) protein kinase, which belongs to phosphatidylinositol 3-kinase-related kinase (PI3K) family. mTOR plays a central role in various cellular processes including protein biosynthesis by controlling transcription, translation and ribosomal biosynthesis thus regulating cell growth and size. Since the mTOR inhibition causes cell cycle arrest at G1 phase, the intervention on mTOR pathway might provide a significant direction to new drug development aimed to the identification of anticancer, antiproliferative, anti-restenosis and immunosuppressor agents. In addition, recent evidence suggests that an altered mTOR pathway is implicated in several neurological disorders [1,2,3].

The mTOR is composed of 2549 amino acidic residues (289 kDa) and organized into five distinct functional domains, (i) 32 replicates called “Huntington, elongation factor 3,PR65/A, TOR” (HEAT) (from amino acid residue 16 to 1345); (ii) “FRAP, ATM, TRRAP” (FAT) (from amino acid residue 1382 to1982); (iii) “FKBP12 rapamycin-binding” region (FRB) (from aminoacid residue 2012 to 2144); (iv) catalytic “kinase domain”(KD) and “regulatory domain” (RD) (from aminoacid residue 2182 to 2516); and (v) “FRAP ATM TRRAP carboxyterminus” (FATC) (from aminoacid residue 2517 to 2549).

From a functional point of view, mTOR exists in two distinct multi-protein complexes commonly known as mTOR complex 1 (mTORC1) and mTOR complex 2 (mTORC2) located in different cellular compartments. It has been well established that the rapamycin-sensitive mTORC1 complex transmits signals of nutrient availability to control various cellular functions, while the rapamycin-insensitive mTORC2 complex regulates the assembly of the cytoskeleton by actin.

**Scheme 1:**
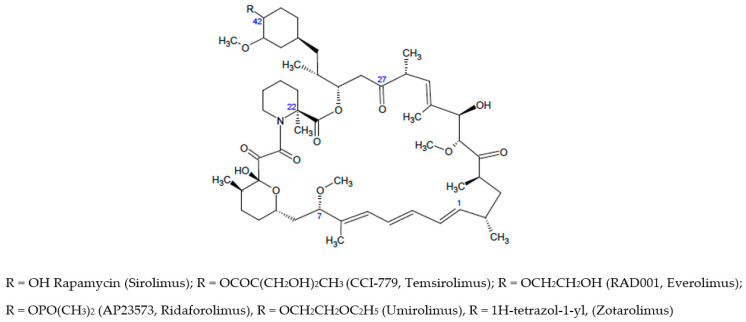
Chemical structures of Rapamycin and rapalogs as well-established mTOR/FKBP12 ligands.

It is well-known that for the microbial-derived macrolide rapamycin (sirolimus) and its analogues (Scheme 1) the inhibition mechanism of mTOR pathway occurs through the binding with FKBP12 domain to form a binary complex; in turn, this complex binds to FRB domain of mTOR only when is associated to other mTORC1 components; this association induces changes in the mTOR conformation, thus inhibiting the crucial kinase activity of the mTORC1 [4,5]. Experimental studies highlighted that in absence of FKBP12, rapamycin possesses a modest affinity for the FRB domain, suggesting that the FKBP12-rapamycin complex plays a pivotal role for the binding to mTOR [6].

Rapamycin is 31-membered macrocyclic lactone produced by *Streptomyces hygroscopicus* which was developed as an immunosuppressant agent as allosteric inhibitor of mTORC1. The crystal structure of the FKBP12–rapamycin–mTOR ternary complex (PDB code 1FAP) unveiled the protein interactions. It has been found that the pipecolyl α-ketoamide of rapamycin anchored it into the proline-binding pocket, whereas the triene system was exposed for interactions with mTOR. Rapamycin displays low water-solubility and poor stability, so that rapamycin analogues (also named rapalogs) with improved biopharmaceutical properties have been developed [7,8] and approved by FDA (see Scheme 1) as the first-generation of mTOR inhibitors to fight cancer malignancies and other diseases. Apart from the weakness in poor druglike properties, the rapalogs possess a complex chemical structure [5]; therefore, the structural modifications of macrolide ring were generally limited.

Further allosteric mTOR inhibitors belonging to rapalog series are modified at C-7, C-22, C-27 and C-42 positions as well as the C-1/C4 fragment. A carefully analysis of structure-activity relationships of rapalogs has been recently reported [5]; the best results were obtained for structural optimization carried out addressing variation at C-42 position leading to FDA approved drugs (see Scheme 1) [5,9,10,11,12,13]. Further modification of rapamycin involved the methoxy substituent bound to C-7 position, thus highlighting the role of this part of macrolide in the interaction with FRB domain [14]. Nelson and coworkers [15] introduced modifications at C-22 and C-27 position, these studies provided newer compounds possessing an improved half-life resulting from (i) the introduction of methyl group (C-22) or (ii) the carbonyl reduction and subsequent acetylation (C-27). Finally, it has been found that rapalogs bearing optimized bulky group (e.g., 1,2-oxazinane ring) at the rapamycin triene moiety (C-1/C-4) might offer neuron survival promotion without immunosoppressive effects [16].

**Figure 1:**
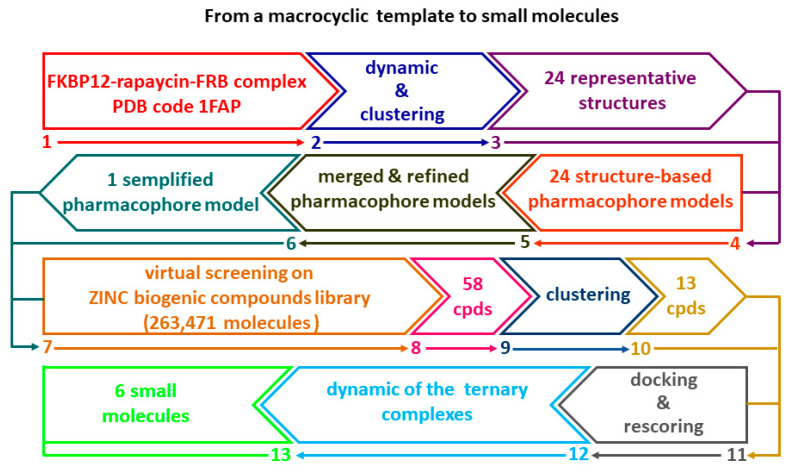
Flowchart illustrating the multistep computational method.

Searching new chemical scaffolds to engender the druglike properties as well as the selectivity of allosteric mTOR inhibitors, an attractive challenge might be the development of chemical entities with reduced molecular weight in which the macrocycle ring does not represent the key structural feature. Based on this assumption, in this study we employed a multistep computational method (Flowchart in Figure 1) to create a structure-based pharmacophoric model as useful tool to discover small molecules as new potential ligands able to form a stable complex with FKBP12 and FRB domain as essential step for the inhibition of mTOR related pathways. It is well known that the generation of structure-based pharmacophore models presents two main limitations: the sensitivity to the atomic coordinates of the system and the number of the pharmacophoric features that can be too low or too high. In this context, MD simulation represents a useful tool to (i) generate multiple sets of coordinates that can be exploited to build multiple pharmacophore models that can be merged in a single model, and (ii) to prioritize features according to their frequency throughout the trajectory [17]. Several studies showed that the integration of protein flexibility into structure-based pharmacophore generation can improve its performance in virtual screening experiments [17,18,19,20,21]. Inspired by these works, we combined MD simulation with pharmacophore modelling in order to explore the most important interactions occurring in the ternary complex FKPB12-rapamicyn-FRB thus unveiling useful hints for the design of small molecules as potential allosteric inhibitors of mTOR activity. For this purpose, this complex was subjected to three independent MD simulations; the resulting frames were clustered according to RMSD, thus obtaining representative conformations of the system that were used to generate multiple structure-based pharmacophore models. The obtained models were merged in one single pharmacophoric hypothesis containing sixteen features that represent a high number for vs. purpose. Therefore, the model was refined basing on the data gained by the three MD simulations and the resulting pharmacophore query was used to screen the ZINC biogenic compounds library. The hits selected from the vs. were docked and rescored by MM-GBSA method leading to a selection of six small molecules whose ability to form a ternary complex with FKPB12 and FRB domain was further investigated by MD simulation. The reported findings could be useful to improve the knowledge for the design of a further generation of effective agents in cancer therapy, as well as in several neuropathies [22].

## 2. Results and Discussion

### 2.1. Molecular Dynamics Simulation

**Figure 2:**
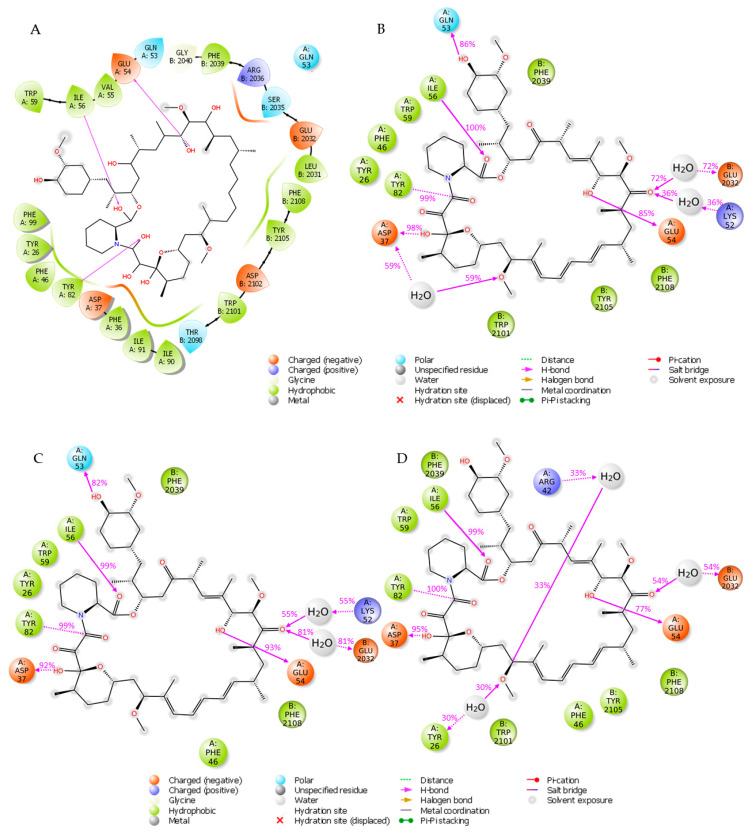
(**A**) Interactions between rapamycin and FKBP12 and FRB from the crystal structure (PDB code 1FAP). (**B**) Interactions occurring during the first MD simulation. (**C**) Interactions occurring during the second MD simulation. (**D**) Interactions occurring during the third MD simulation.

In the first round of our study, we in depth analyzed the FKBP12-rapamycin-FRB ternary complex and explore the interactions occurring between rapamycin and its two protein partners. To perform this study, we started from the crystal structure accessible on RCSB Protein Data Bank archive with entry 1FAP [4]. As depicted in Figure 2A, the analysis of the crystallographic data unveils that rapamycin interacts with FKBP12 through (i) three H-bond interactions with E54, I56 and Y82 and (ii) a network of hydrophobic contacts with Y26, F36, F46, V55, I56, W59, Y82, I90, I91 and F99. In addition to the described interactions, two more H-bonds involving Q53 and D37 of FKBP12 were reported in literature [23]. Instead, the rapamycin establishes solely hydrophobic interactions with FRB domain of mTOR through residues W2101, Y2104, Y2105, F2108, L2031 and F2039 confirming the previously published data [4].

To evaluate the stability of the above-mentioned interactions we carried out three independent 25 ns molecular dynamics (MD) simulations by using the software Desmond [24]. The root mean square deviation (RMSD) for the two protein domains and ligand rapamycin was computed and plotted as reported in Figure S1 of Supplementary Materials. Specifically, in the first two simulations both the protein system and the ligand exhibited a stable behavior (Figure S1A,B in Supplementary Materials), while in the last trajectory an increase of rapamycin RMSD was observed at 5 ns; then, it remains stable for the rest of the trajectory (Figure S1C in Supplementary Materials). The interactions occurred between the ligand and the two protein partners were analyzed to get more insights about the contacts occurring more frequently during the three simulations. Specifically, for each trajectory we examined the interactions that appear more than 30% of the simulation time (Figure 2B–D). The obtained results were compared with the protein-ligand contacts arising from the crystal structure depicted in Figure 2A of FKBP12-rapamycin-FRB ternary complex (PDB code 1FAP). The three MD simulations revealed that the H-bonds involving I56 and Y82 from FKBP12 were very stable throughout the three trajectories appearing with a frequency ≥99%, while the one involving E54 occurred with frequencies of 85%, 93% and 77% in the first, second and third simulation, respectively. Moreover, through MD simulation we retrieved the H-bond interactions involving (i) D37 which appeared more than 90% over the three trajectories, and (ii) Q53 which occurred more than 80% of the simulation time in the first two trajectories and less than the 30% in the third one. Concerning the hydrophobic interactions, the most stable involved W59 of FKBP12 and F2039 and F2108 residues from FRB domain, occurring more than 50% of the simulation time; in details Figure S2 contains a histogram plot reporting the overall protein-ligand contacts occurring during each of the MD trajectories.

### 2.2. Pharmacophore Modelling

**Figure 3:**
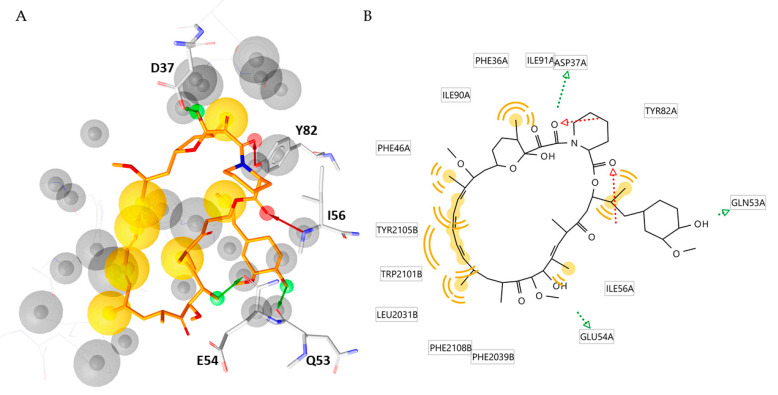
(**A**) Structure-based pharmacophore model derived from the X-ray structure of the FKBP12-rapamycin-FRB ternary complex (PDB code 1FAP). Rapamycin is represented as orange sticks. Pharmacophoric features are color coded: yellow spheres represent hydrophobic features; green arrows represent H-bond donors and red arrows H-bond acceptors. The residues involved in H-bond are highlighted as grey sticks (**B**) 2D depiction of the interactions described by the pharmacophore model. The aminoacidic residues involved in such interactions are highlighted.

In the second round of this study, the MD simulation results were exploited to generate a helpful and simplified structure-based pharmacophore model deciphering the peculiar interactions of the FKBP12-rapamycin-FRB ternary complex. To achieve this objective, the three-dimensional hypothesis was generated by using the software LigandScout V4.4.2 [25]. As displayed in Figure 3, the obtained model was constitued by twelve features (i) two H-bond acceptors, (ii) three H-bond donors, (iii) seven hydrophobic features and twentysix exclusion volumes. The model suggested that the three H-bond donors pointed to D37, E54 and Q53, while the two H-bond acceptors involved the residues Y82 and I56.

To increase our understanding of interactions in the FKBP12-rapamycin-FRB ternary complex we included the structural information derived from our MD simulations in the obtained three-dimensional models. Thus, the frames resulted from each trajectory were clustered based on RMSD to get representative conformations of the system. The clustering process, performed by the Desmond Trajectory Clustering tool implemented in Maestro suite, yielded nine clusters for the first simulation, seven for the second and third for the third one. For each cluster a representative structure was identified and used to create structure based-pharmacophore models that are displayed in Supplementary Materials. To our satisfaction, the seven hydrophobic features and the H-bond acceptor involving Ile56 found in the X-ray model were reconfirmed in all the generated pharmacophore models. The H-bond donors involving D37 and E54 and the H-bond acceptor involving Y82 were observed in 20/24 models, while the H-bond donors involving Q53 was found in 17/24 models. Additionally, a H-bond acceptor involving Y26, was detected in 18/24 representative frames.

**Figure 4:**
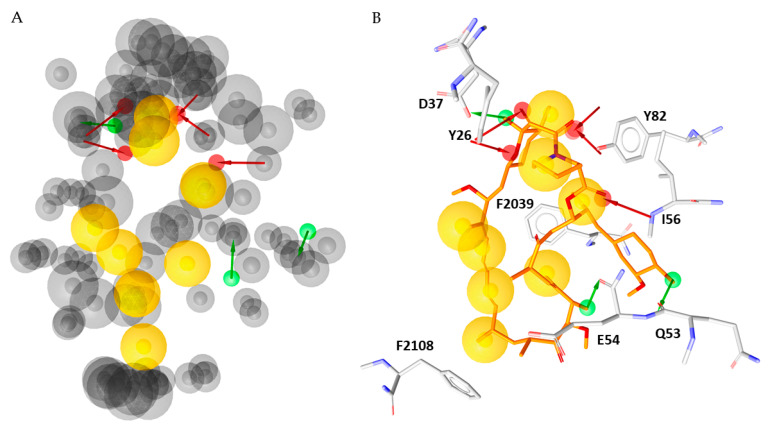
(**A**) Merged pharmacophore model created by the models generated from the representative snapshots of each cluster. (**B**) Rapamycin aligned with the merged pharmacophore model.

Taken together, these data furnished a merged dynamic pharmacophore; the resulting model is displayed in Figure 4, it contains 8 hydrophobic features, 5 H-bond acceptors, three H-bond donors and eighty-six exclusion volumes.

At this point in our computational study, we prioritized several of the obtained features, thus simplifying the merged pharmacophore model; the assessment of the relevance of several key features was based on their occurrence in the previously generated structure-based pharmacophore models, as well as on the percentage of the corresponding interactions in the three MD simulations. Regarding the H-bonds features, we maintained the H-bond acceptor with I56 found in all the generated pharmacophore models. Instead, we excluded the H-bond donor involving Q53 and the H-bond acceptor involving Y26 found solely in models 17 and 18, respectively. The remaining H-bond acceptor formed with Y82 and H-bond donors with D37 and E54 were found in 20 models. To avoid too restrictive a pharmacophore model, we decided to discard the H-bond donor involving E54 that emerged to be less stable over the three MD simulations if compared to the H-bond with D37 and Y82 (see Figure 1 and Figure S2). Finally, the two H-bond acceptor features, that arise from the interaction between the carbonyl of the amide group of rapamycin and Y82, were interpolated in one single feature and all the features vectors were converted in spheres.

**Figure 5:**
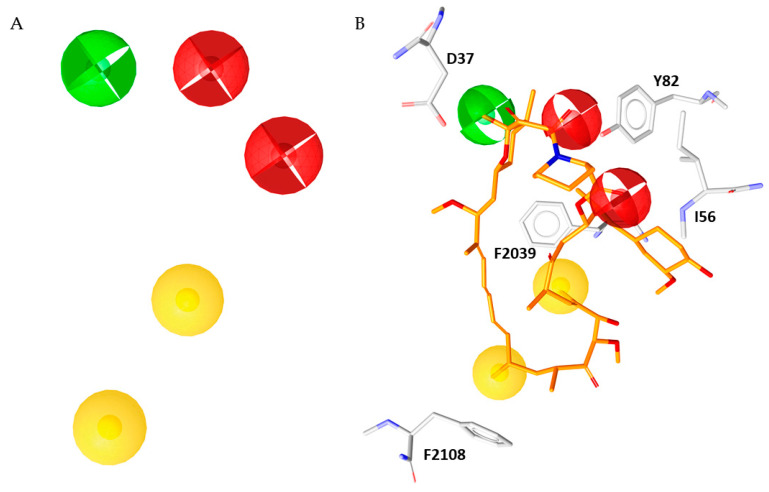
(**A**) Simplified five-features pharmacophore model. (**B**) Rapamycin (orange sticks) superimposed to the simplified pharmacophore model.

Concerning the hydrophobic features, we kept in mind the concept that LigandScout software follow a rather unspecific criteria to place hydrophobic features respect to the more specific restrains adopted for H-bond donors and acceptors. Indeed, small variations on the geometry of the ligand and/or the protein could markedly affect the classification of an acceptor-donor pair as hydrogen bond or not, while it could have a less influence on hydrophobic features [17]. Taken together these considerations, we decided to maintain two hydrophobic features that represent the two crucial interactions engaged with F2039 and F2108, which revealed to be the most stable in the three MD simulations (see Figure S2). On the basis of previous selection criterion, a newer and very simplified pharmacophore model was obtained; it now consisted of two hydrophobics, two H-bond acceptors and one H-bond donor features (Figure 5). As result, the canonical bifunctional binding mode of rapamycin was guaranteed by the five features involving the crucial hydrophobic and hydrophilic moieties depicted in Figure 5B.

### 2.3. Virtual Screening

The above described simplified model was used to screen the ZINC [26] biogenic compounds library collecting 263,471 compounds from natural sources, obtaining 58 hits (the chemical structures of all compounds are reported in Supporting Materials in Table S4). In order to select compounds structurally different, the resulting molecules were clustered according to their similarity applying the Pharmacophore RDF-code similarity measure as implemented in LigandScout software. Thirteen clusters were identified, in turn, from each cluster the compound that showed the highest Pharmacophore Fit-Score was selected. As result, we collected 13 structurally different compounds (Table S5) to perform the subsequent steps of our in silico study.

### 2.4. Molecular Docking, Rescoring and MD Simulation

The binding mode of the 13 hits selected through the virtual screening procedure was analyzed by molecular docking by means of Glide software [27,28,29]. Initially, the rapamycin was docked in the same binding site to validate our protocol [30]; the obtained rapamycin poses were rescored by MM/GBSA method and the orientation possessing the lowest predicted ΔG_bind_ value was chosen for the analysis; in this way, the docking pose was in good agreement with the crystallographic binding conformation with a resulting RMSD value of 1.806 Å, as shown in Figure S3 of Supplementary Materials.

**Figure 6:**
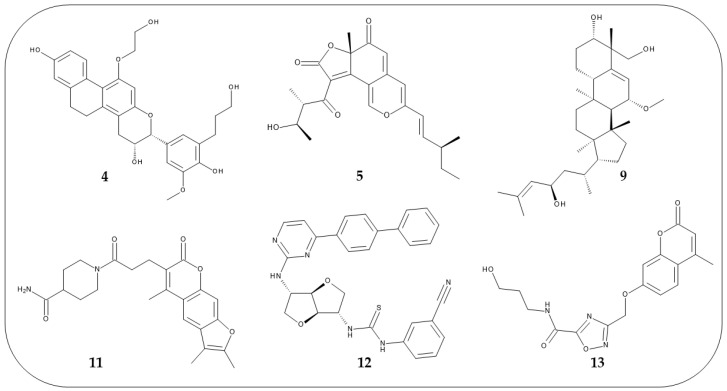
2D structures of the six compounds selected from the docking studies.

The same protocol was applied to the hits selected from the virtual screening and were selected as potential mTOR inhibitors the six compounds (Figure 6) that showed a ΔG_bind_ value lower than −74.00 kcal/mol (see Table S6). A collection of predicted parameters and bioactivity is reported in Supplementary Materials, these in silico data suggested that compounds **4**, **5**, **9**, **11**–**13** might be chemical templates for further structural modification (Table S7A–C). For these selected compounds, additional chemical details are reported in Table S8.

**Figure 7:**
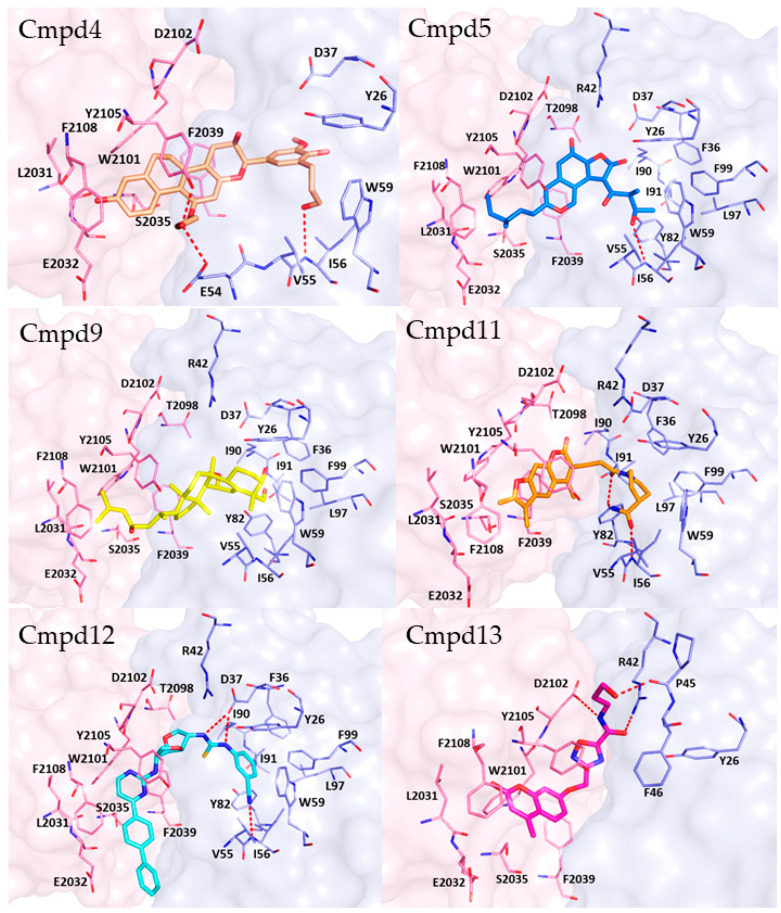
Plausible binding mode for each selected compound (**4**, **5**, **9**, **11**, **12**, or **13**). The residues of FRB domain and FKBP12 are highlighted respectively as pink and light blue sticks. Each compound is represented in sticks. H-bonds are represented by red dashed lines. The image was created by using PyMOL 2.4.0 19/05/2020 software (https://pymol.org/(accessed on 19 May 2020)).

The plausible binding mode for each selected compound (**4**, **5**, **9**, **11**, **12**, or **13**) is displayed in Figure 7. Analogously to rapamycin, all the compounds might bind the FRB domain (colored in pink in Figure 7) of mTOR occupying the hydrophobic pocket lined by L2031, W2101, Y2105, F2039 and F2108. Additionally, compounds **4** and **13** might interact with the FRB domain establishing H-bonds with Y2105 and D2102, respectively. Hydrophobic interactions were observed for W2101 and compounds **11**, **12** and **13**, as well as for Y2105 and compounds **11** and **13**. Furthermore, compound **11** established pi-pi interaction with F2039.

Concerning the interactions with FKBP12 (colored in light blue in Figure 7), compounds **4**, **5**, **11** and **12** and formed H-bonds with I56, while H-bonds with E54 and Y82 might be engaged by compounds **4** and **11**, respectively. Instead, compound **13** could bind to FKBP12 by forming H-bonds with R42 and P45. Furthermore, hydrophobic contacts were observed for compounds **5**, **9**, **12** and W59, I56 and F99. Finally, aromatic interactions were detected between compound **12** and F99.

Notably, all the selected hits engaged profitable contacts with the amino acid residues crucial for the binding of rapamycin to FKBP12 and FRB domain (Figure S4 in Supplementary Materials) and, therefore, they could act as allosteric mTOR inhibitors with a mechanism similar to that of rapamycin or rapalogs currently in therapy.

In the last step of our work, the stability of the ternary complexes obtained from the docking studies for all selected compounds was probed by running 25 ns MD simulation by means of Desmond software. For this purpose, the RMSD related to protein backbone and each ligand was monitored throughout the simulation as plotted in Figure S5 in Supplementary Materials. The results highlighted that all the compounds could form a stable ternary complex with the two proteins FKBP12 and FRB domain and this represents the mechanistic basis for the inhibition of mTOR activity.

## 3. Materials and Methods

### 3.1. Molecular Dynamics Simulation and Clustering

Molecular dynamics simulations were performed by using the software Desmond [9] employing the crystallographic structure of the FKPB12-rapamycin-FRB ternary complex retrieved from RCSB Protein Data Bank (PDB code 1FAP). Hydrogen atoms and bond orders were assigned to the protein by the Protein Preparation Wizard [31] implemented in Maestro V 12.5.139 [32]. The system was solvated in an orthorhombic box using TIP3P water model and neutralized by adding 7 Cl^−^ ions by means of “System Builder” tool. A salt concentration of 0.15 M of NaCl was included in the simulation box to reproduce the physiological conditions and the minimize volume option was turned on. The simulation was set up through the “Molecular dynamics” tool. The system was relaxed before the simulation by using the protocol implemented in Desmond and then it was simulated for 25 ns by NTP ensemble at 310 K and 1 atm. The OPLS3e force field was employed to parametrize the entire molecular system [33]. The coordinates were saved every 25 ps resulting in 1000 frames. Three independent runs were performed for the X-ray complex by using random seeding. The obtained trajectories were clustered according to RMSD employing “Desmond Trajectory Clustering”, setting a frequency value of 20 and using up to 10 clusters. The MD simulations of the complexes obtained from the docking studies were executed employing the aforementioned protocol.

### 3.2. Pharmacophore Modelling and Virtual Screening

LigandScout software V 4.4.2 was used for pharmacophores generation and virtual screening. The representative conformations of the system obtained from the clustering process were used as starting points for the construction of structure-based pharmacophore models. The resulting models were aligned and merged to obtain a unique pharmacophore model by using the option “Merge pharmacophore models and interpolate overlapping features” implemented in LigandScout. The virtual screening was performed by setting the option “Get the best matching conformation” as retrieval mode. The hits resulting from the virtual screening were clustered according to the “Pharmacophore RDF-code similarity” measure present in LigandScout software.

### 3.3. Docking and Rescoring

Docking studies were performed by using Glide software employing the 3D coordinates of the ternary complex FKBP12-rapamycin-FRB prepared as described in paragraph 3.1. The ligands were prepared by using the LigPrep [34] tool implemented in Maestro, generating the possible ionization states at pH 7.0 ± 2.0 by using Epik [35,36] and retaining the specified chirality. The receptor grid was automatically generated by selecting the co-crystallized ligand (rapamycin). In particular, Glide software uses two boxes to perform the calculation: an outer box that defines the volume in which the grid potentials are computed and an inner box which defines the volume that the ligand center explores during the search. The dimensions of the outer box calculated for this structure were 27.539 × 27.539 × 27.539 Å, while for the inner box the default dimensions, 10 × 10 × 10 Å, were maintained. Both boxes have center −8.777, 26.997, 36.484 (see Table 1).

A maximum of ten poses per ligand were generated and the “post docking minimization” option was turned on. The docking simulation was performed in extra precision (XP) mode without constraints. MM/GBSA rescoring was carried out by means of Prime MM/GBSA tool implemented in Schrödinger suite using the VSGB solvation model and the minimize sampling method. Protein residues were treated as flexible applying the “using constraints on flexible residues” option. For each compound, the pose with the lowest ΔG_bind_ value was selected for the analysis and representation.

## 4. Conclusions

This work describes a multistep structure-based approach aimed to identify new inhibitors targeting mTOR kinase at rapamycin binding site. We collected information about the structural features essential for the interaction of FKBP12-rapamycin complex with FRB domain of mTOR that could be exploited for the design of allosteric mTOR inhibitors, the small molecules being a valuable alternative in respect to the rapalogs that possess low druglike properties.

## Figures and Tables

**Table 1:** Protocols used for molecular docking studies.

Receptor	Native Ligand	Coordinates of Grid Center	Dimensions of the Outer Box (Å)	Dimensions of the Inner Box (Å)
FKBP12-FRB domain (PDB code 1FAP)	Rapamycin	X = −8.777 Y = 26.997 Z = 36.484	X = 27.539 Y = 27.539 Z = 27.539	X = 10 Y = 10 Z = 10

## Data Availability

Data sharing not applicable.

## Supplementary Materials

The following are available online, Figure S1: RMSD plots related to three MD simulations of the x-ray complex 1FAP, Figure S2: Histogram plot of the protein-ligand contacts occurring during the three MD simulation of the x-ray complex 1FAP, Table S1: Structure-based pharmacophore models and related 2D interaction scheme of the representative frames obtained from the clustering of simulation I, Table S2: Structure-based pharmacophore models and related 2D interaction scheme of the representative frames obtained from the clustering of simulation II, Table S3: Structure-based pharmacophore models and related 2D interaction scheme of the representative frames obtained from the clustering of simulation III, Table S4: 2D structure of the 58 hits obtained from virtual screening, Table S5: Ligand-pharmacophore mapping and Pharmacophore-Fit score of the hits selected from the virtual screening, Figure S3: Docking pose of rapamycin superimposed to its crystallographic binding conformation within the x-ray complex 1FAP, Table S6: Binding free energy values obtained by MM/GBSA rescoring, Table S7: In silico prediction of the drug-like properties the selected compounds **4**, **5**, **9**, **11**–**13**, Table S8: Smile strings and CAS number of the selected compounds **4**, **5**, **9**, **11**–**13**, Figure S4: Superimposition of the docking poses of compounds **4**, **5**, **9**, **11**–**13** with the crystallographic binding conformation of rapamycin, Figure S5: RMSD plots related to the MD simulations of the ternary complexes obtained from the docking and rescoring studies.
