# Supplementary material for: In Silico Strategy for Targeting the mTOR Kinase at Rapamycin Binding Site by Small Molecules
Source: Molecules. 2021 Feb 19;26(4):1103. doi: 10.3390/molecules26041103 (PMC7922000; doi:10.3390/molecules26041103)
Supplement: Supplementary file 1 [file molecules-26-01103-s001.pdf]

In Silico Strategy for Targeting the mTOR Kinase at Rapamycin Binding Site by Small Molecules

Serena Vittorio [1], Rosaria Gitto [1], Ilenia Adornato [1], Emilio Russo [2] and Laura De Luca [1,*]

[1] Department of Chemical, Biological, Pharmaceutical and Environmental Sciences, University of Messina, Viale Palatucci 13, I-98168, Messina, Italy; svittorio@unime.it (S.V.); rgitto@unime.it (R.G.); ilenia_adornato@hotmail.it (I.A.); ldeluca@unime.it (L.D.L.)

[2] Science of Health Department, School of Medicine, University "Magna Graecia" of Catanzaro, Viale Europa e Germaneto, 88100 Catanzaro, Italy; erusso@unicz.it (E.R.)

Corresponding: ldeluca@unime.it; Tel.: +39-090-676-6410 (L.D.L.)

Table of contents

FigureS1 RMSD plots related to three MD simulations of the x-ray complex 1FAP……………..…S2

FigureS2 Histogram plot of the protein-ligand contacts occurring during the three MD simulation of the x-ray complex 1FAP……………………………………………………………………...S4

TableS1 Structure-based pharmacophore models and related 2D interaction scheme of the representative frames obtained from the clustering of simulation I..S6

TableS2 Structure-based pharmacophore models and related 2D interaction scheme of the representative frames obtained from the clustering of simulation II………………………………….S9

TableS3 Structure-based pharmacophore models and related 2D interaction scheme of the representative frames obtained from the clustering of simulation III……………………………..…S12

TableS4 2D structure of the 58 hits obtained from virtual screening…………………………..….S15

TableS5 Ligand-pharmacophore mapping and Pharmacophore-Fit score of the hits selected from the virtual screening…………………………………………………………………...….…S19

FigureS3 Docking pose of rapamycin superimposed to its crystallographic binding conformation within the x-ray complex 1FAP……………………………………………………….....S22

TableS6 Binding free energy values obtained by MM/GBSA rescoring…………………….……....S23

Tables S7A-C *In silico* prediction of selected physicochemical parameters, lipophilicity, solubility, drug-likeness, pharmacokinetic profile, toxicity and bioactivity for compounds **4, 5, 9, 11-13**.........S24

TableS8 Smile strings and CAS number of the selected compounds **4**, **5**, **9**, **11-13**………………....S26

FigureS4 Superimposition of the docking poses of compounds **4**, **5**, **9**, **11-13** with the crystallographic binding conformation of rapamycin………………………………………………S27

FigureS5 RMSD plots related to the MD simulations of the ternary complexes obtained from the docking and rescoring studies……………………………………………………………...S28

FigureS1

A) MD SIMULATION I

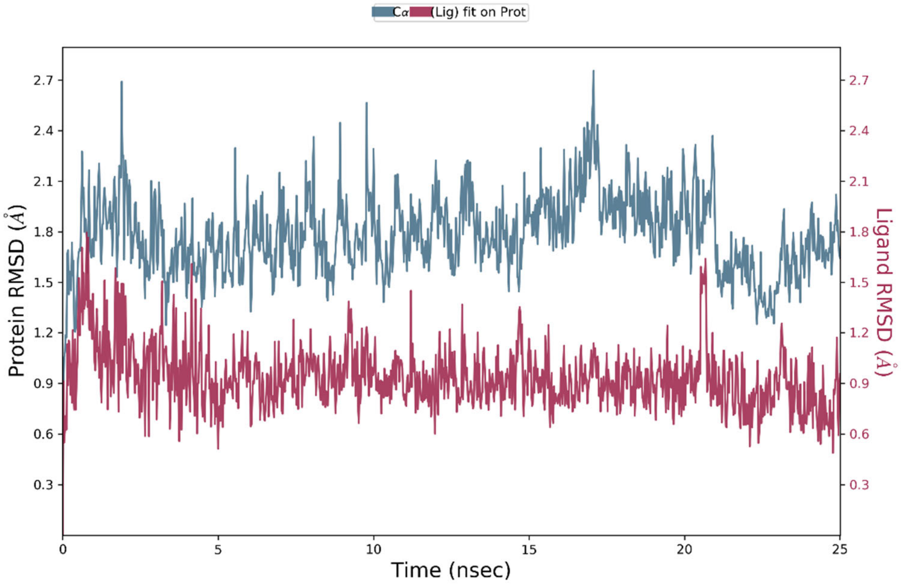

B) MD SIMULATION II

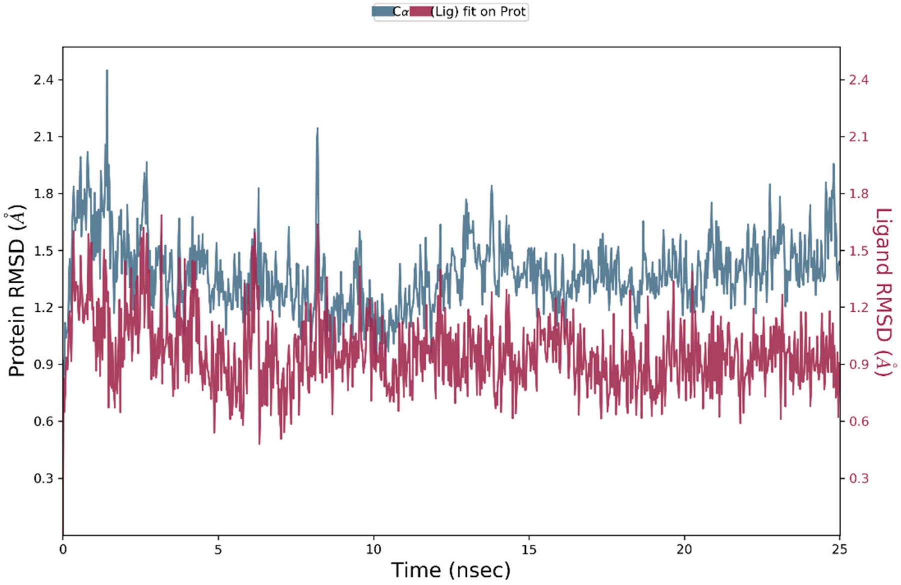

C) MD SIMULATION III

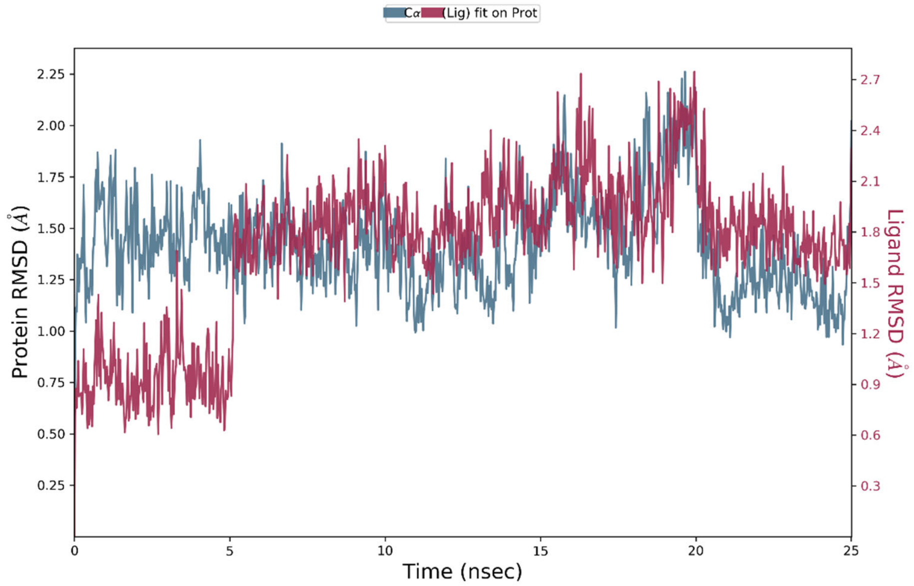

Figure S1 RMSD plots of Cα of the protein portion (blue) and rapamycin (red) related to the three MD simulations.

FigureS2

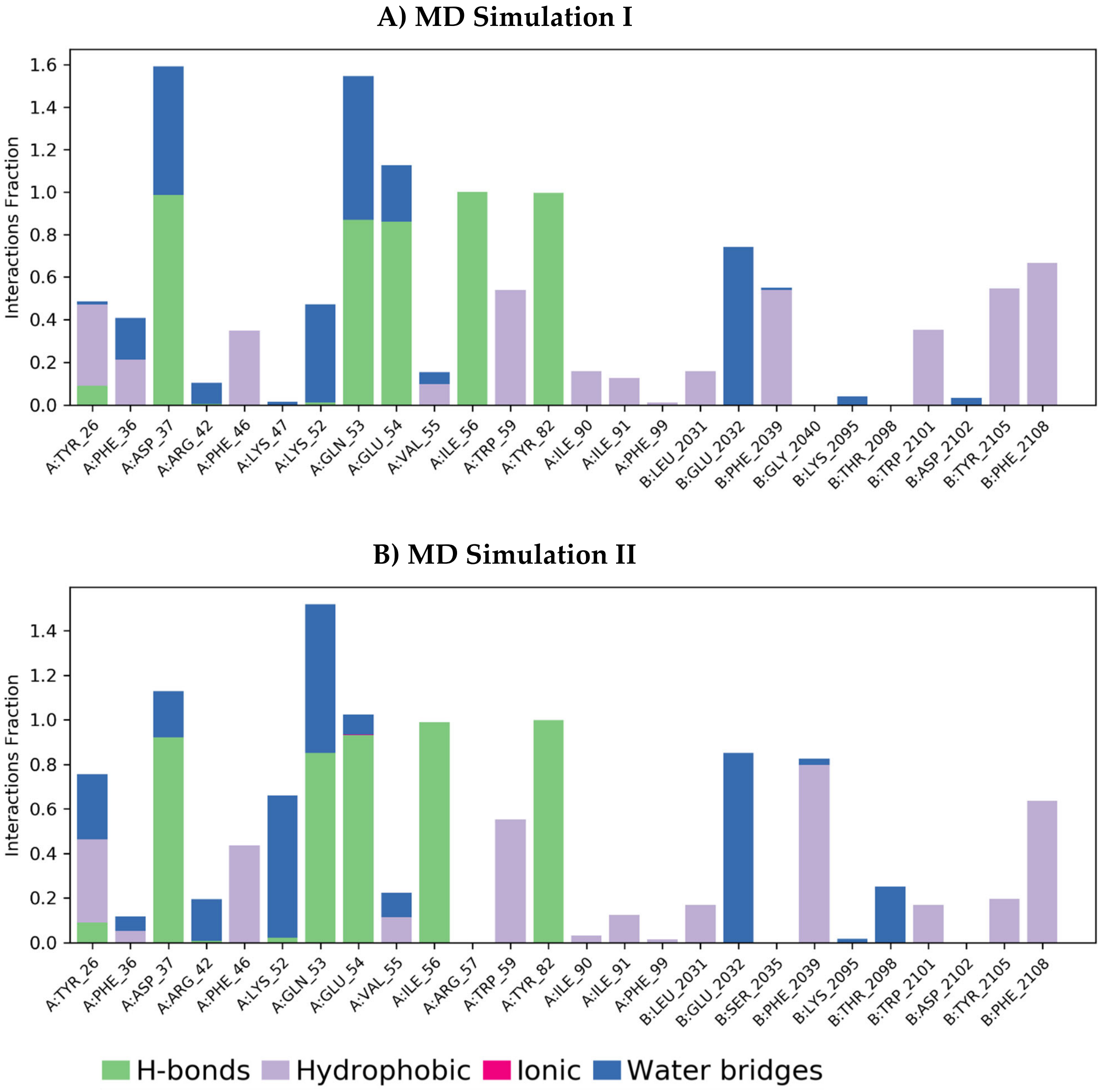

A) MD Simulation I
Interactions Fraction
0.0
0.2
0.4
0.6
0.8
1.0
1.2
1.4
1.6
A:TYR_26
A:PHE_36
A:ASP_37
A:ARG_42
A:PHE_46
A:LYS_47
A:LYS_52
A:GLN_53
A:GLU_54
A:VAL_55
A:ILE_56
A:TRP_59
A:TYR_82
A:ILE_90
A:ILE_91
A:PHE_99
B:LEU_2031
B:GLU_2032
B:PHE_2039
B:GLY_2040
B:LYS_2095
B:THR_2098
B:TRP_2101
B:ASP_2102
B:TYR_2105
B:PHE_2108
B) MD Simulation II
Interactions Fraction
0.0
0.2
0.4
0.6
0.8
1.0
1.2
1.4
A:TYR_26
A:PHE_36
A:ASP_37
A:ARG_42
A:PHE_46
A:LYS_52
A:GLN_53
A:GLU_54
A:VAL_55
A:ILE_56
A:ARG_57
A:TRP_59
A:TYR_82
A:ILE_90
A:ILE_91
A:PHE_99
B:LEU_2031
B:GLU_2032
B:SER_2035
B:PHE_2039
B:LYS_2095
B:THR_2098
B:TRP_2101
B:ASP_2102
B:TYR_2105
B:PHE_2108
H-bonds
Hydrophobic
Ionic
Water bridges

C) MD Simulation III

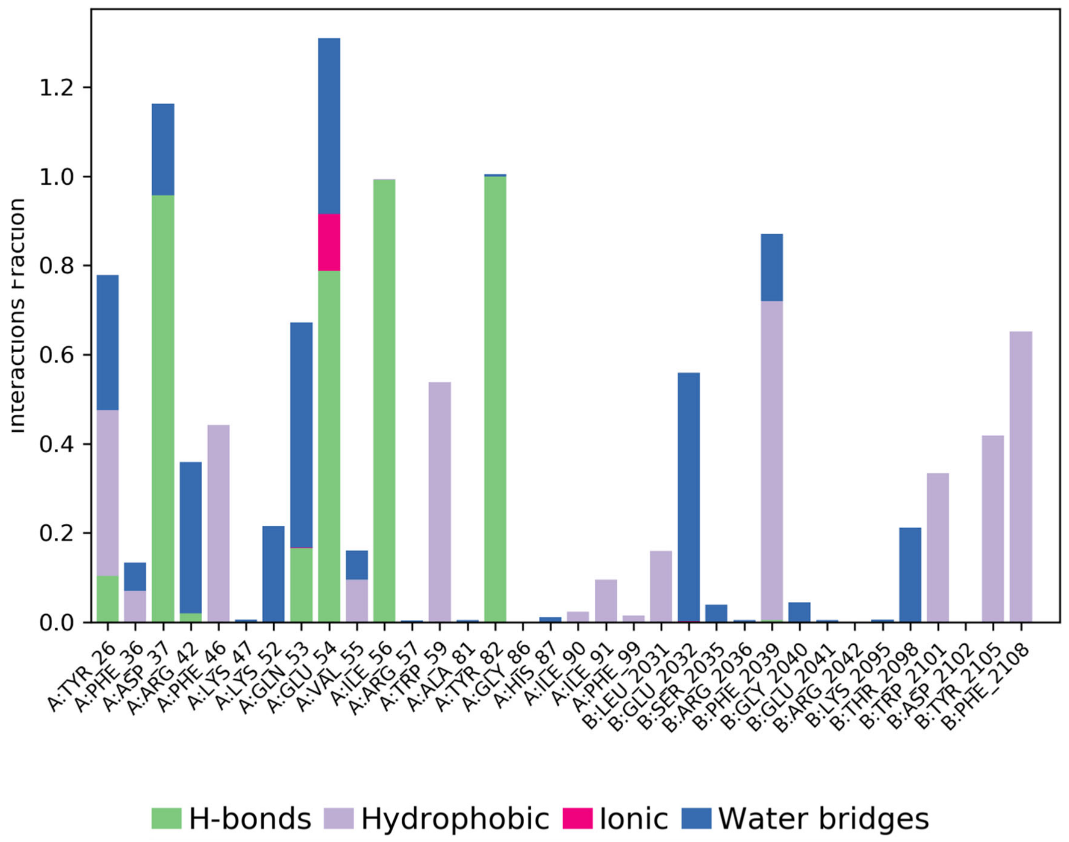

FigureS2. Protein-ligand contacts over the three MD simulation. In the x axis, the residues involved in the interactions are reported, while the frequency with which a given interaction appears during the simulation is reported in the y axis (For instance, a value of 0.7 suggests that the specified interaction is present for the 70% of the simulation time. Values over 1 are possible because some protein residues can establish multiple contacts with the ligand).

Table S1

<table>
<tr><th colspan="3">Simulation I</th></tr>
<tr><td>Cluster 1</td><td>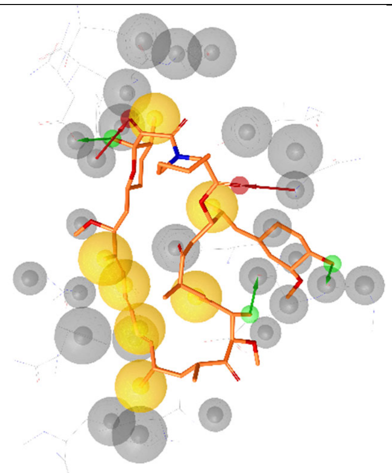</td><td>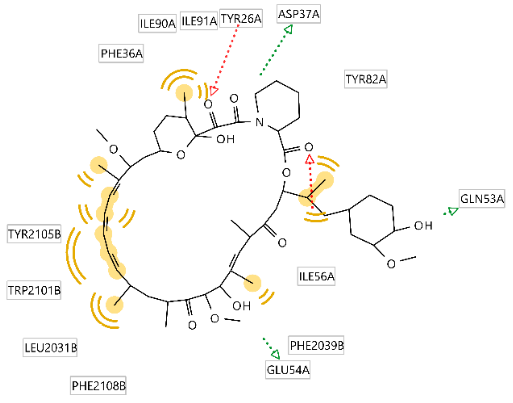
</td></tr>
<tr><td>Cluster 2</td><td>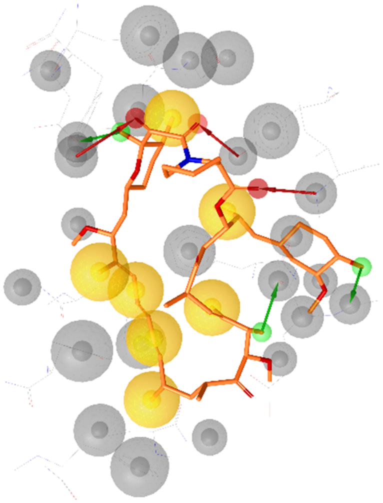</td><td>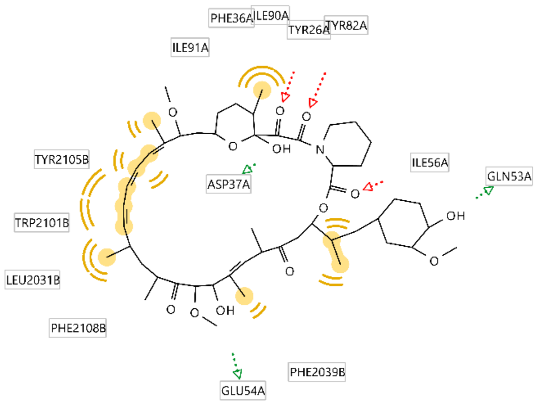
</td></tr>
<tr><td>Cluster 3</td><td>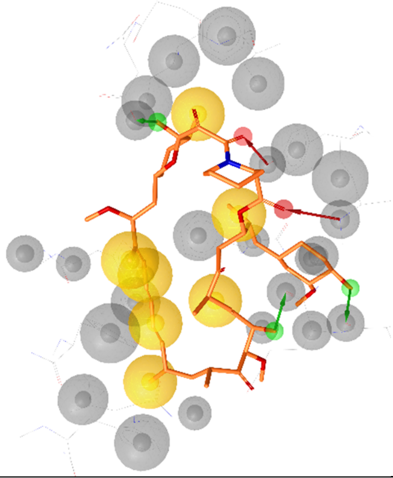</td><td>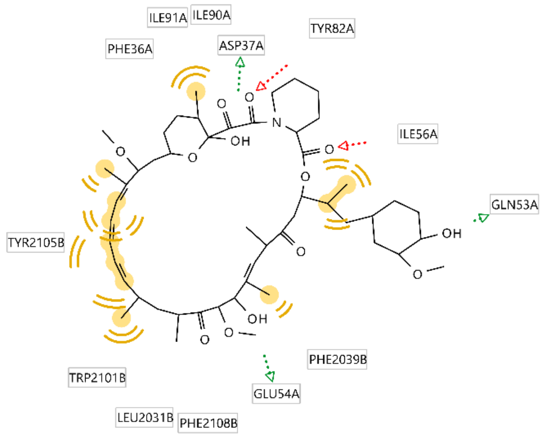
</td></tr>
</table>

Cluster 4

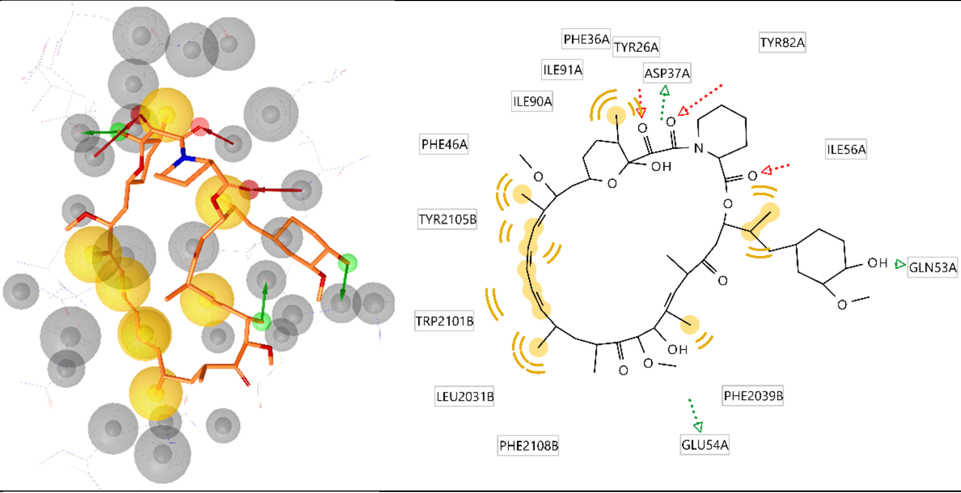

Cluster 5

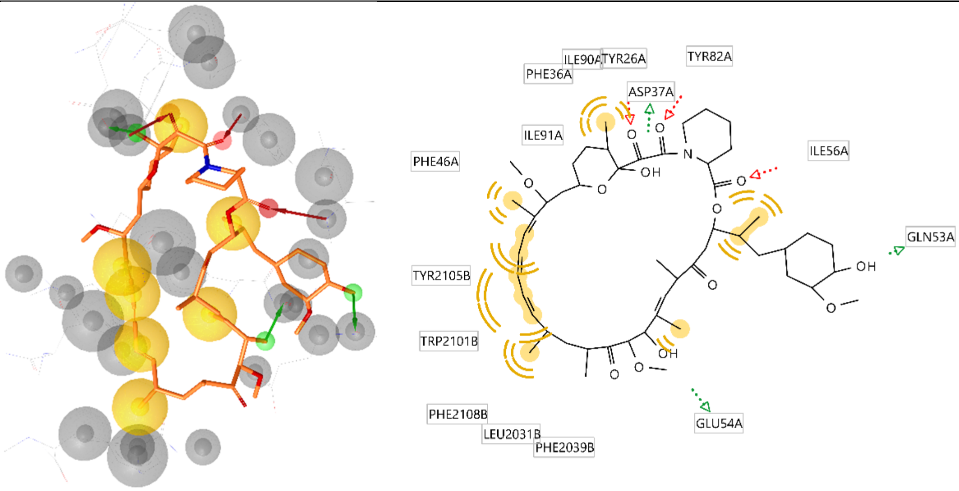

Cluster 6

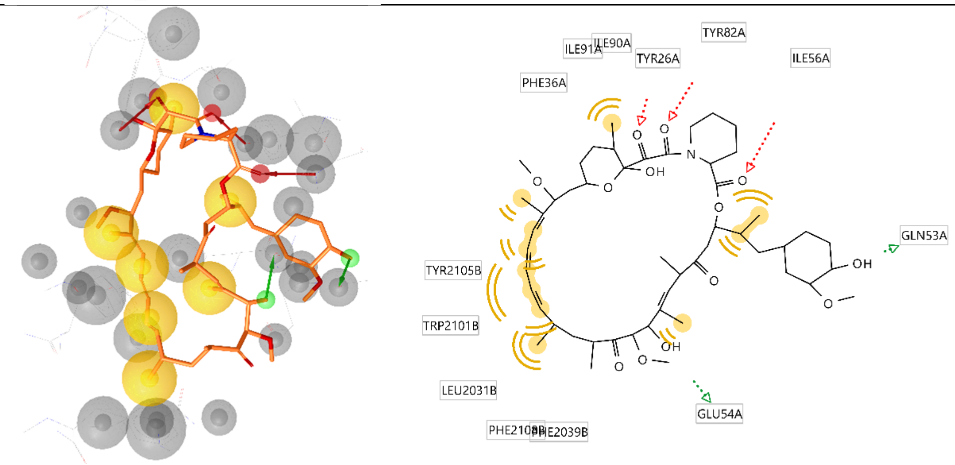

Cluster 7

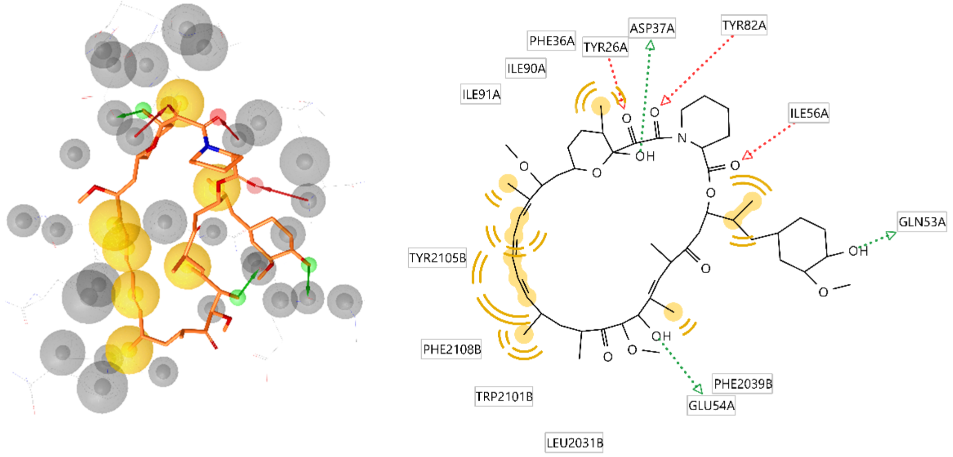

Cluster 8

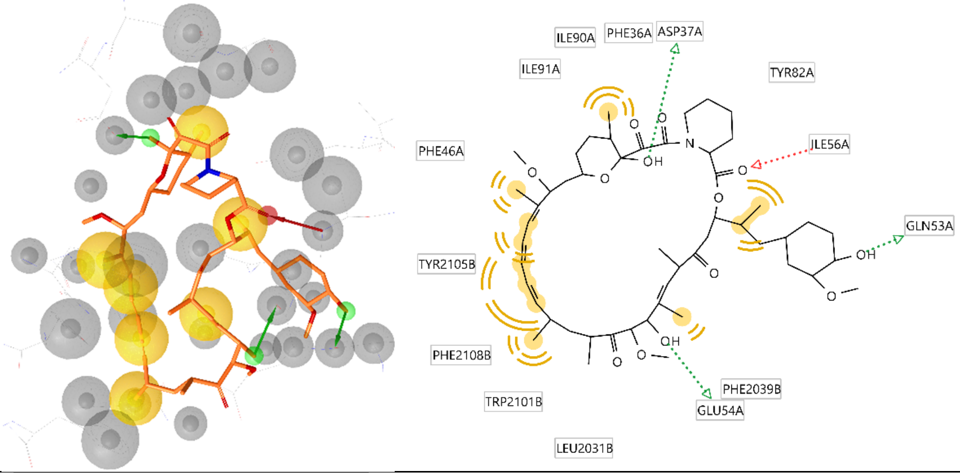

Cluster 9

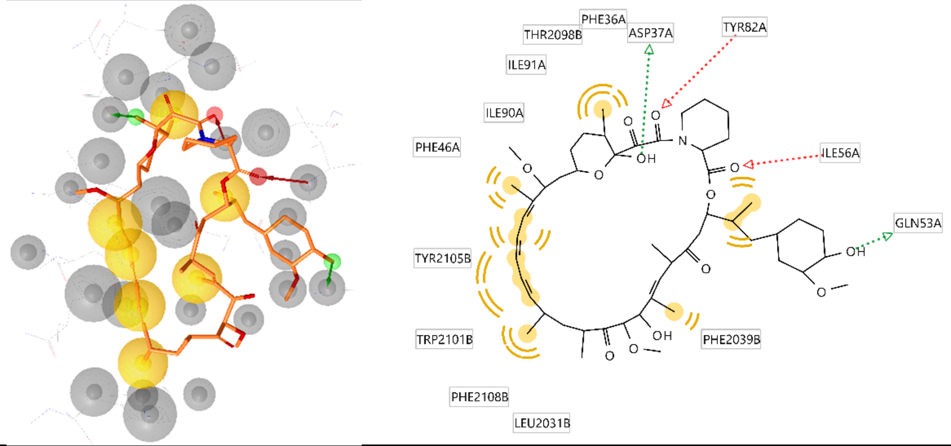

Table S2

	Simulation II	
**Cluster 1**	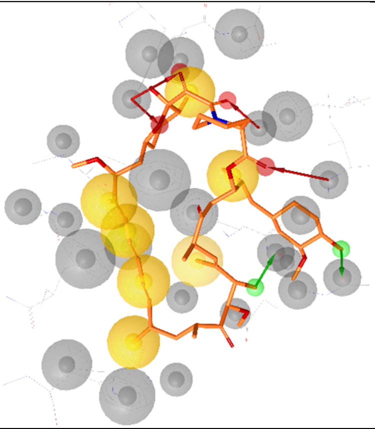	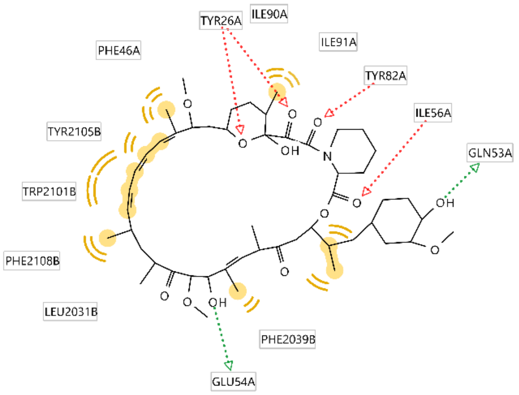
**Cluster 2**	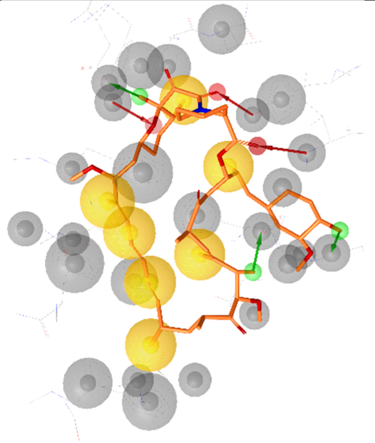	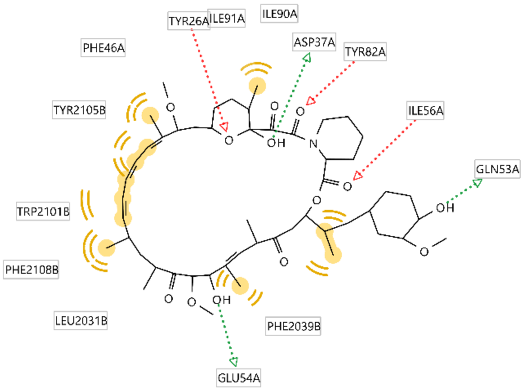
**Cluster 3**	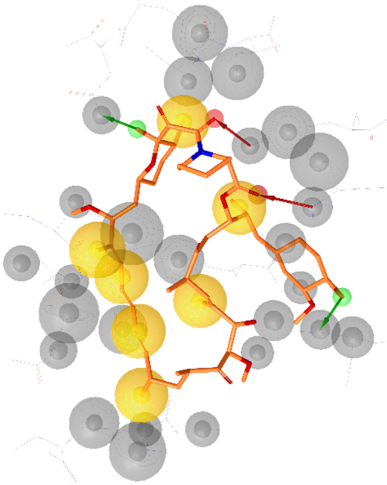	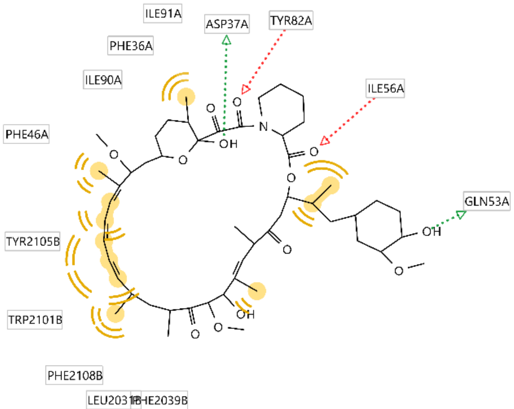

Cluster 4

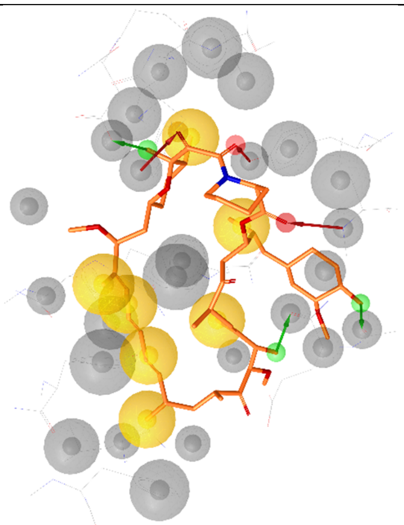

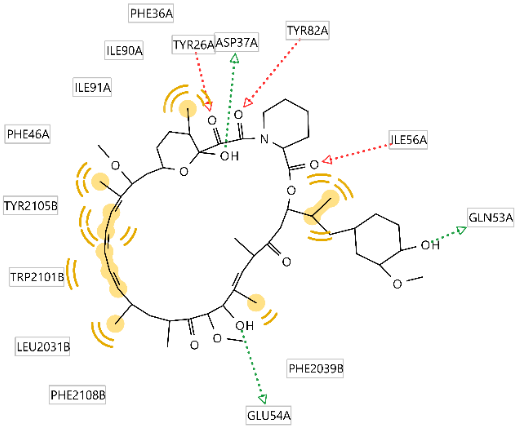

Cluster 5

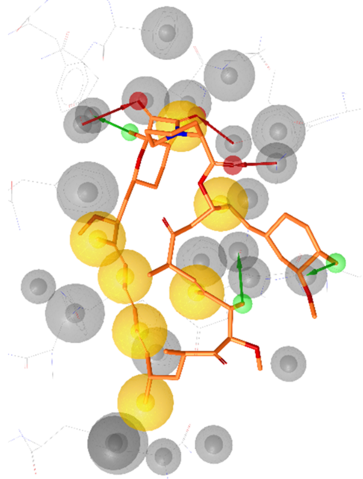

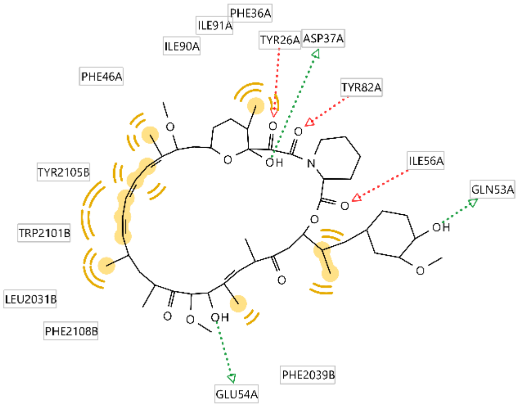

Cluster 6

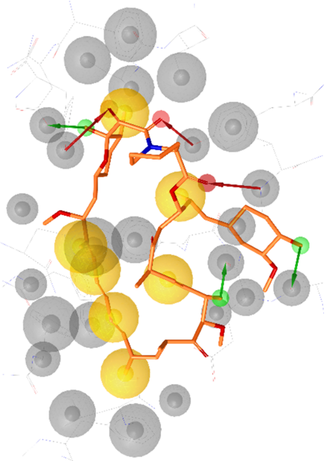

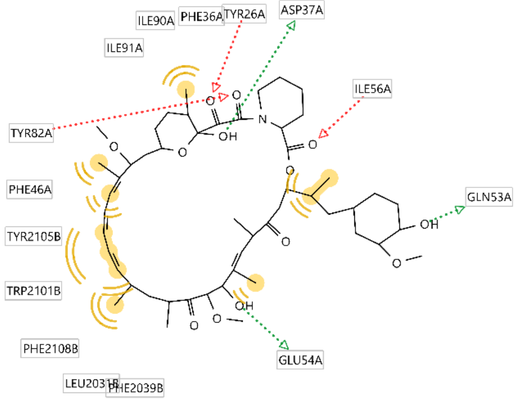

Cluster 7

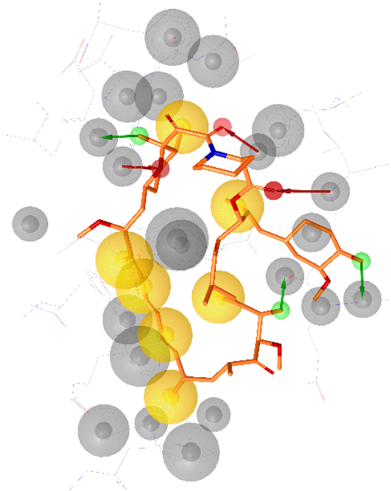

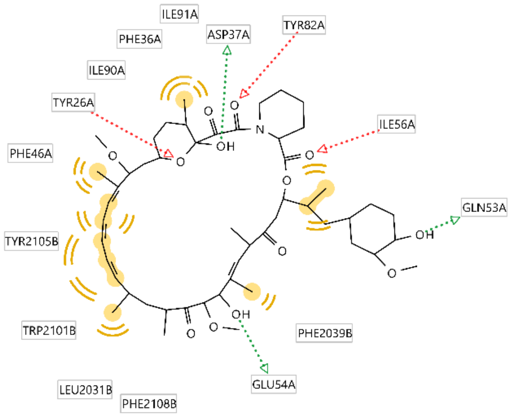
ILE91A
PHE36A
ASP37A
TYR82A
ILE90A
TYR26A
ILE56A
PHE46A
GLN53A
TYR2105B
TRP2101B
PHE2039B
GLU54A
LEU2031B
PHE2108B

Table S3

Simulation III

Cluster 1

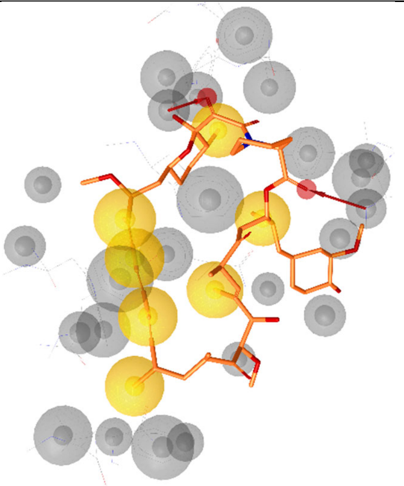

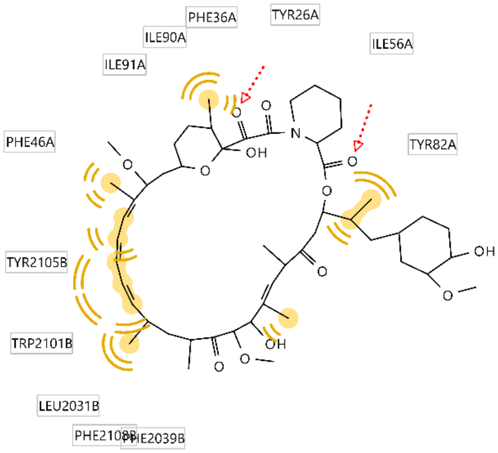

Cluster 2

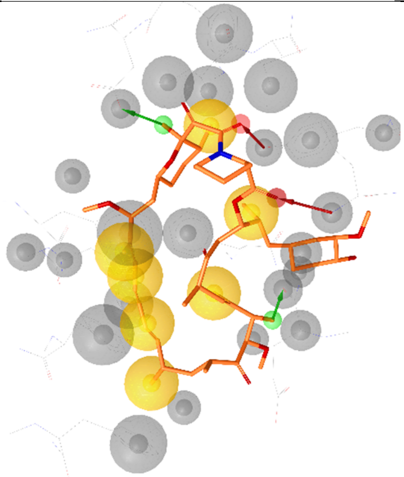

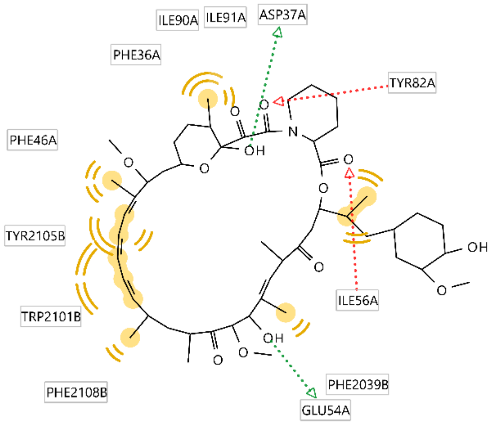

Cluster 3

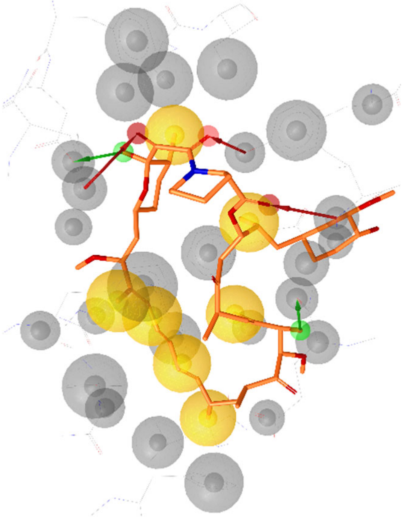

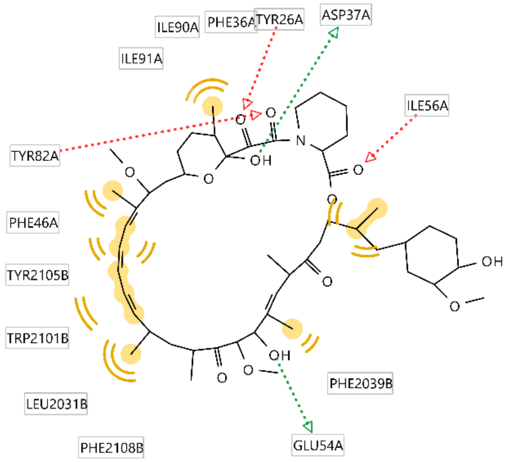

Cluster 4

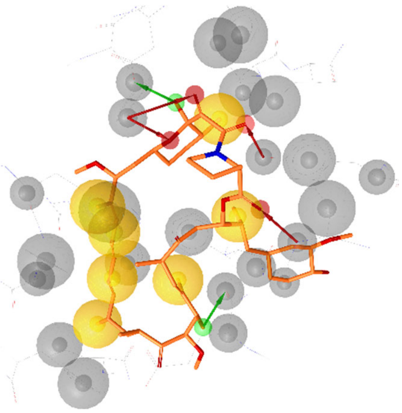

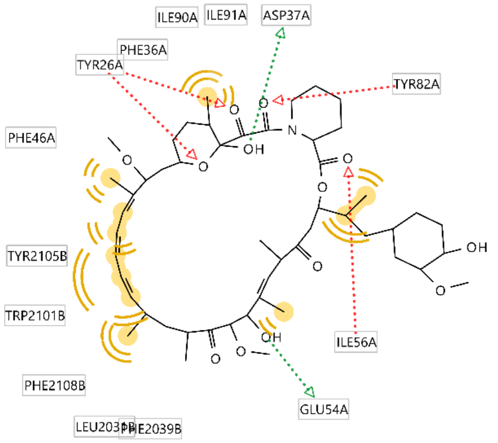

Cluster 5

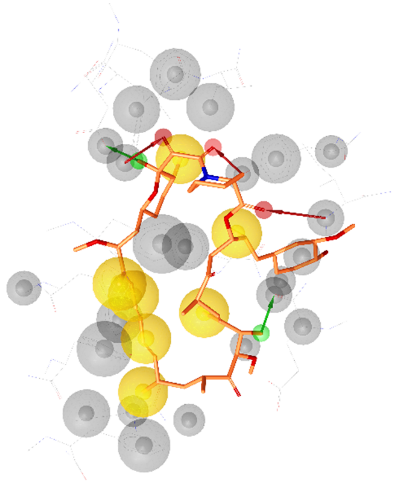

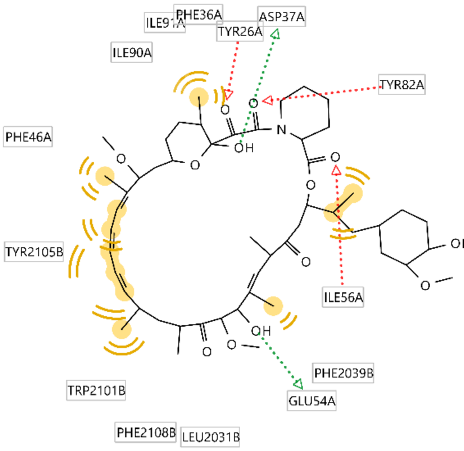

Cluster 6

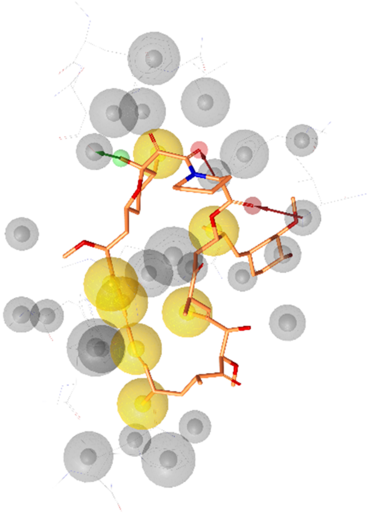

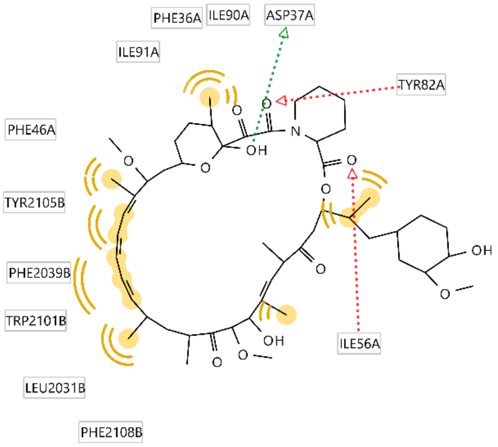

**Cluster 7**	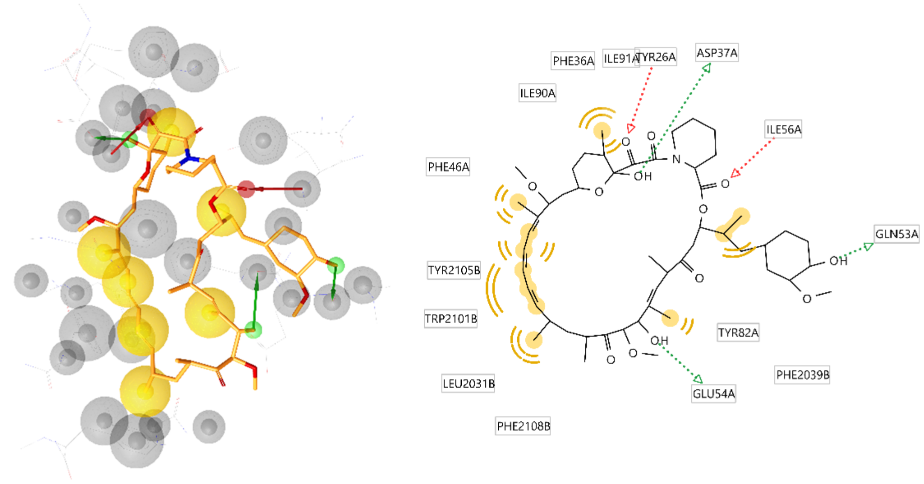
**Cluster 8**	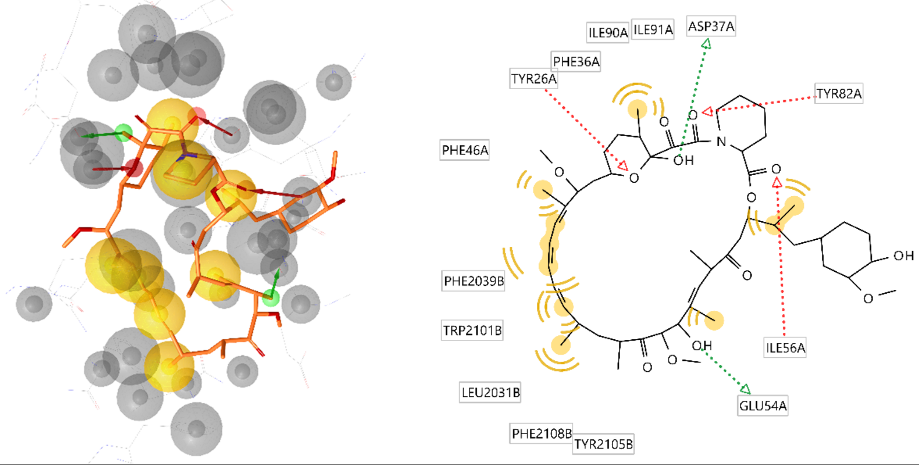

TableS4

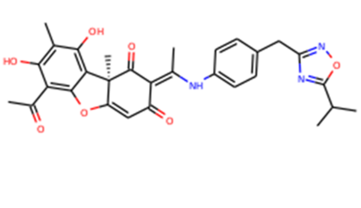 ZINC822285042	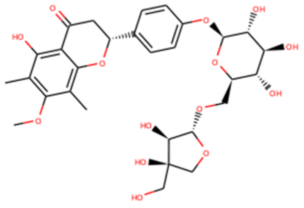 ZINC514288263	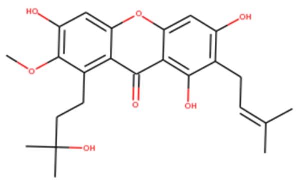 ZINC14727633
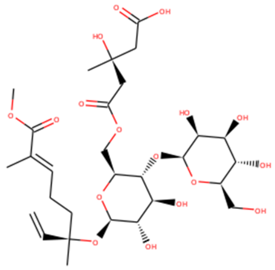 ZINC253534442	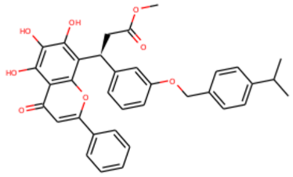 ZINC108448918	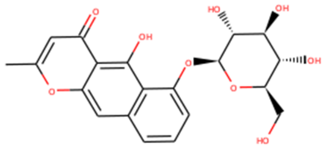 ZINC6069586
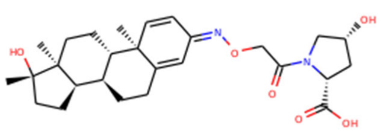 ZINC253533111	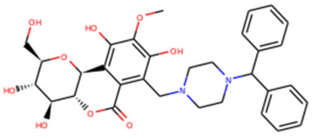 ZINC79193178	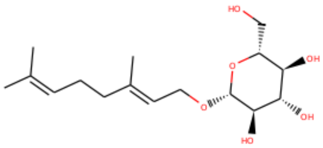 ZINC13515322
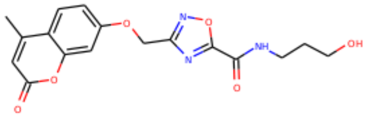 ZINC20728457	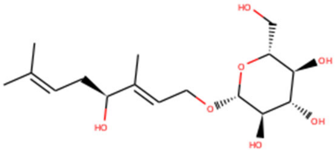 ZINC31169747	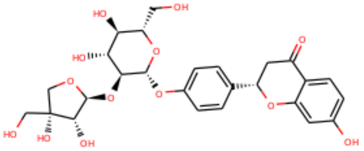 ZINC253529539
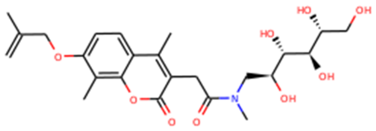 ZINC8764219	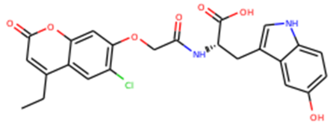 ZINC2128675	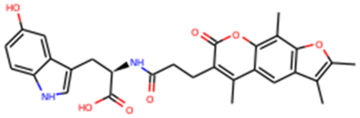 ZINC12874177
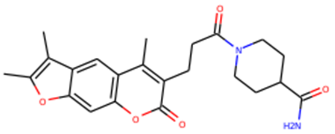 ZINC2111213	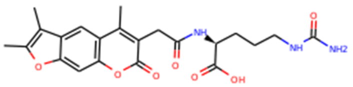 ZINC2100872	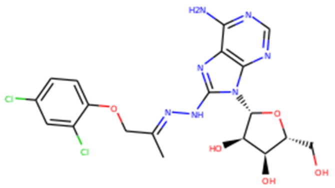 ZINC8918560

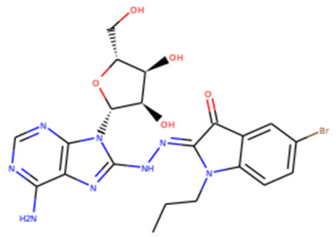

ZINC100753838

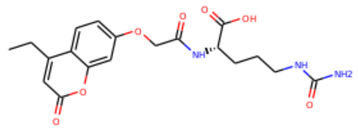

ZINC2146401

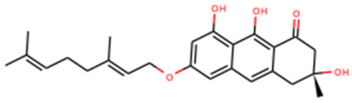

ZINC4098466

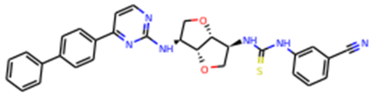

ZINC5399642

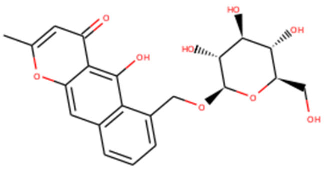

ZINC33830106

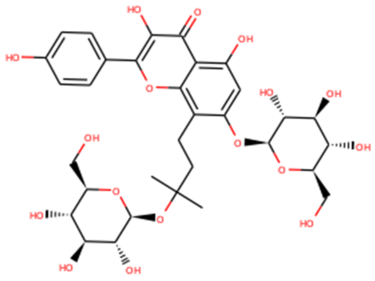

ZINC95914302

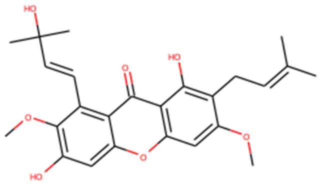

ZINC14727640

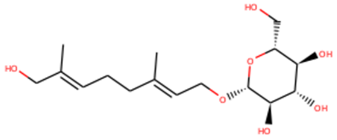

ZINC34353277

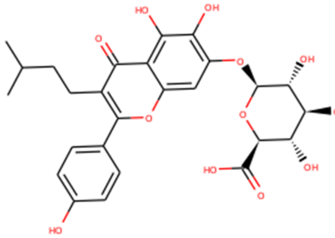

ZINC85507133

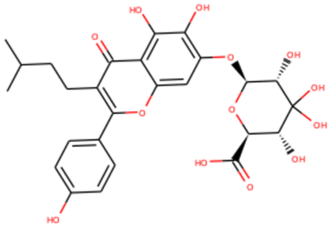

ZINC85507119

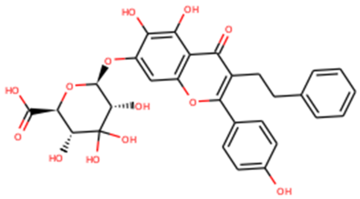

ZINC85507071

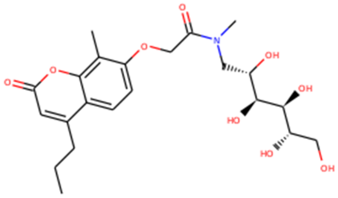

ZINC12892517

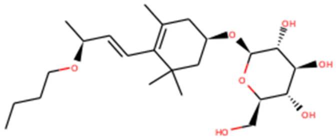

ZINC523042390

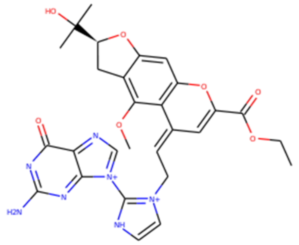

ZINC103528669

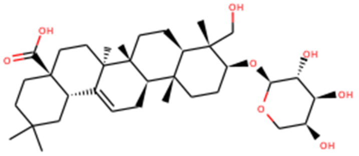

ZINC8234236

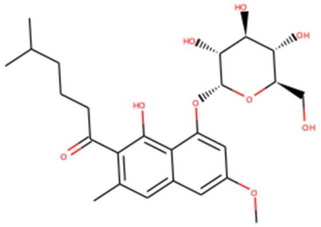

ZINC85592508

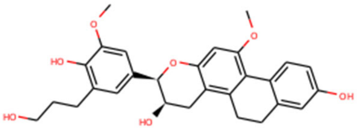

ZINC85568227

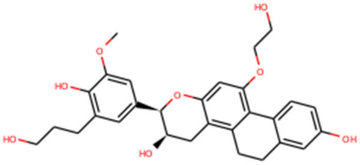

ZINC85568190

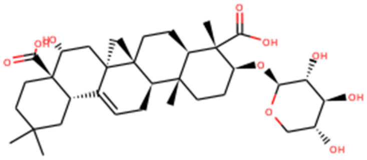

ZINC67913308

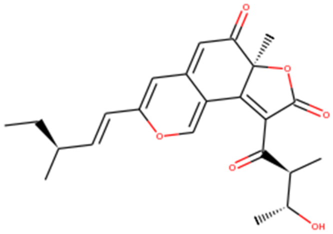

ZINC72109671

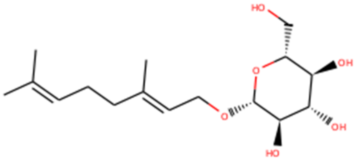

ZINC13515322

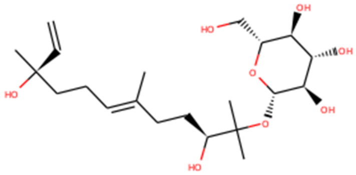

ZINC35466048

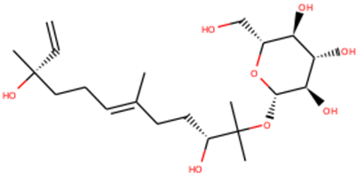

ZINC35050063

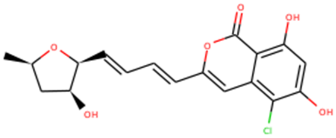

ZINC35464494

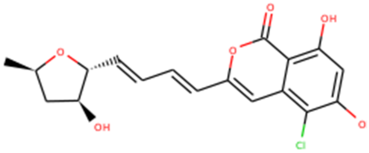

ZINC31168686

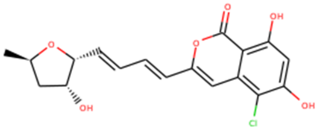

ZINC31168678

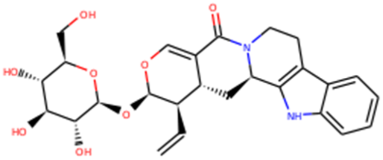

ZINC38139467

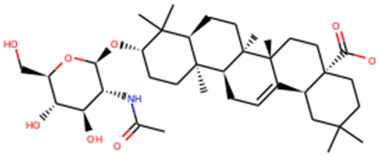

ZINC253407195

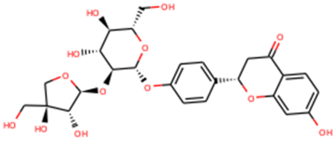

ZINC253529536

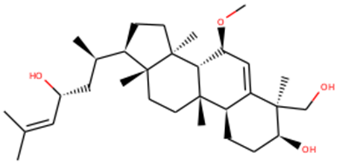

ZINC49793204

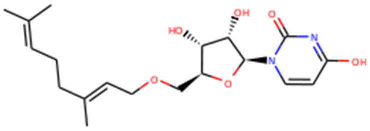

ZINC253534170

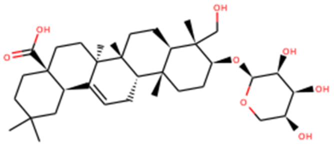

ZINC95099386

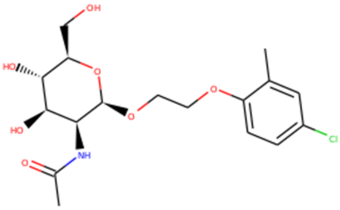

ZINC241738595

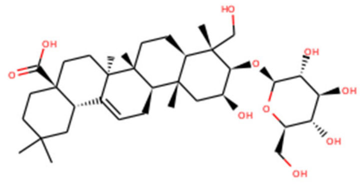

ZINC255287498

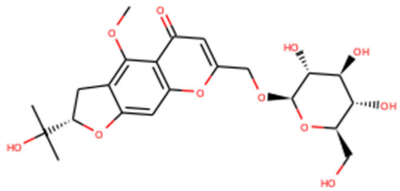

ZINC31158236

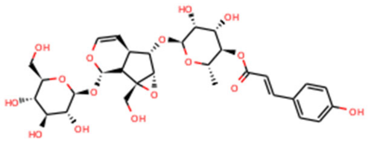

ZINC253389700

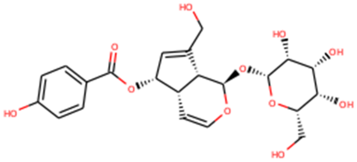

ZINC35455270

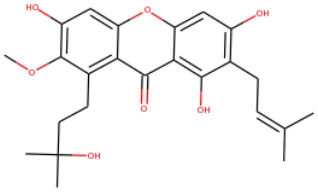

ZINC14727633

Table S5

Cmpd	Fit-Score	Cluster	Structure	Pharmacophore Mapping
**1**	56.92	13	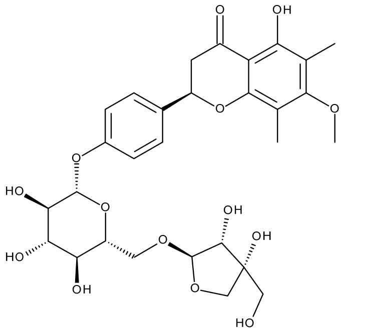	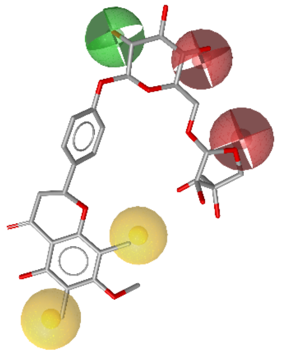
**2**	56.80	11	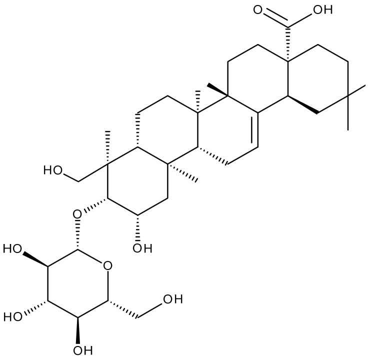	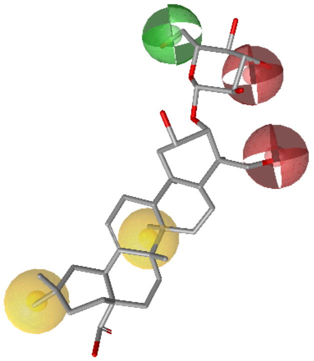
**3**	56.43	12	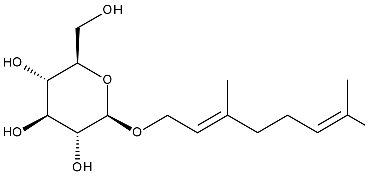	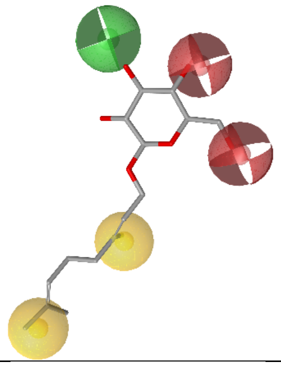
**4**	56.39	10	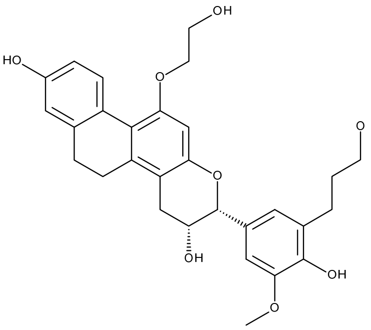	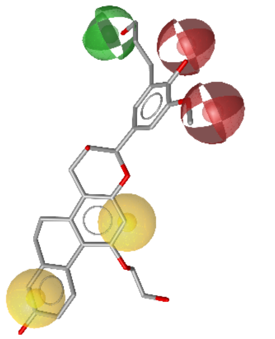

**5**	56.27	3	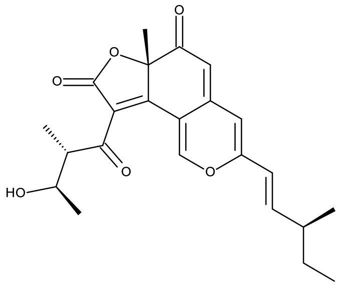	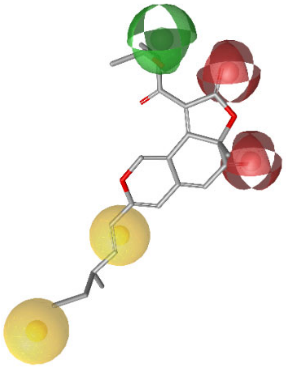
**6**	56.06	8	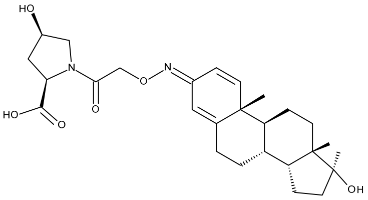	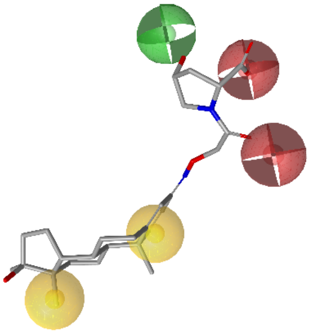
**7**	56.02	4	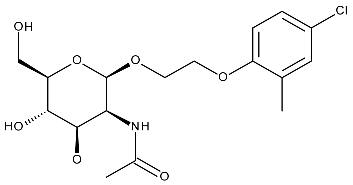	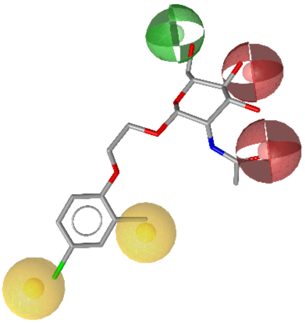
**8**	55.82	9	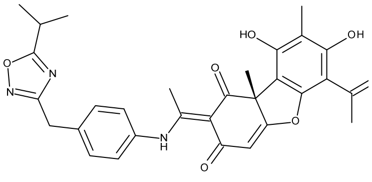	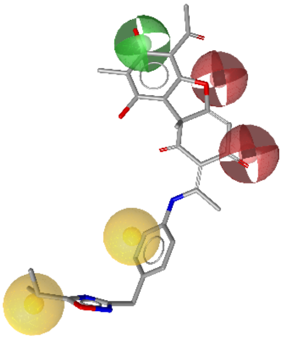
**9**	55.81	2	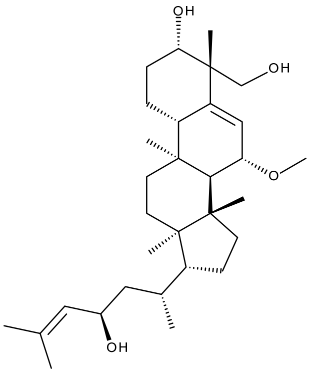	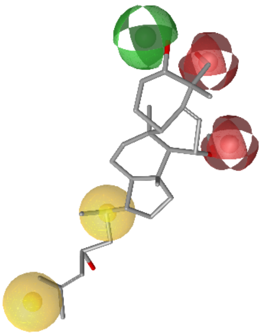

**10**	55.50	7	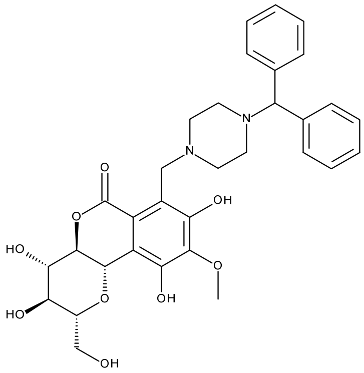	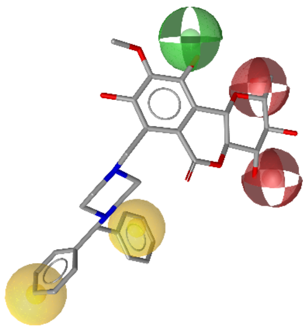
**11**	55.48	5	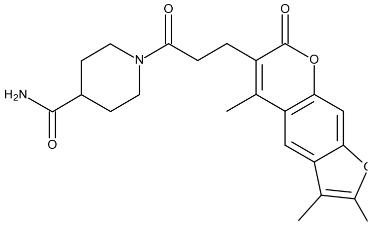	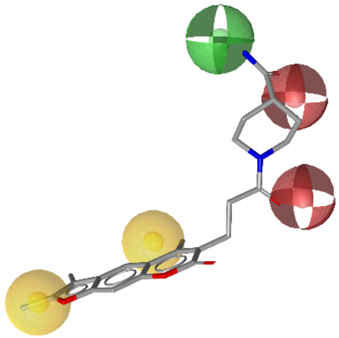
**12**	55.46	1	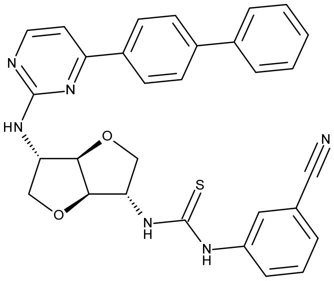	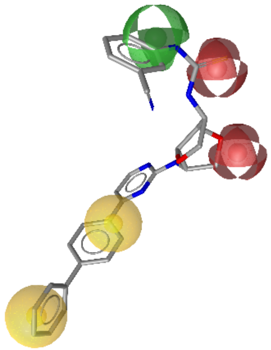
**13**	55.25	6	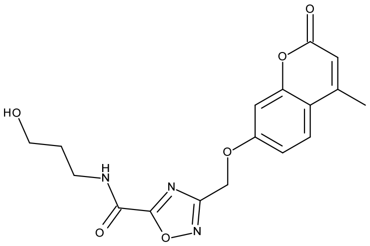	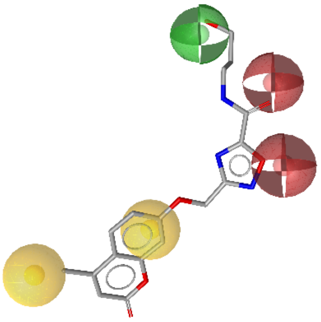

Figure S3

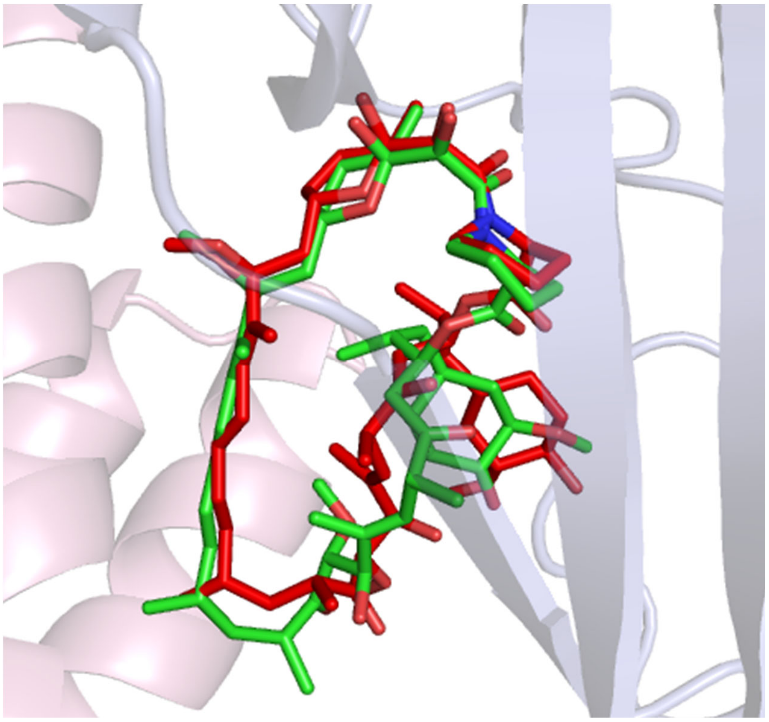

Figure S3. Docking pose of rapamycin (green sticks) superimposed to its crystallographic pose (red sticks). The RMSD value, calculated setting the x-ray position as reference, is 1.806Å. The image was created by using PyMOL software (https://pymol.org/).

Table S6

Cmpd	ΔG_{bind} (kcal/mol)
**1**	-71.45
**2**	-56.61
**3**	-50.07
**4**	-83.10
**5**	-74.79
**6**	-47.18
**7**	-45.53
**8**	-68.70
**9**	-75.25
**10**	-69.11
**11**	-74.25
**12**	-79.24
**13**	-75.81
**Rapamycin (x-ray)**	-169.47
**Rapamycin (docking)**	-124.07

The Tables S7A, S7B, and S7C collect the computational prediction of *s*elected physicochemical parameters, lipophilicity, solubility, drug-likeness, pharmacokinetic profile, toxicity and bioactivity for compounds **4, 5, 9, 11-13**. The prediction was performed through Pre-ADMET ((https://preadmet.bmdrc.kr), Molinspiration(https://molinspiration.com), SwissADME (http://swissadme.ch/), and QikProp v.6.5 (Schrödinger Release 2020-4: QikProp, Schrödinger, LLC, New York, NY, 2020.)

TableS7 (A) *S*elected physicochemical parameters, lipophilicity solubility and drug-likeness for compounds **4**, **5**, **9**, **11-13** predicted by SwissADME, molinspiration and QikProp tools

entry	MW*	TPSA*	TPSA**	PSA#	iLogP*	**m**iLogP**	QPlogP# (octanol/ water)	Water Solubility*	Lipinski*	Ghose*	PAINS*
**4**	508.56	128.84	128.84	127.82	3.64	3.21	3.208	Moderately soluble	Yes<br>1 violation: MW>500	No<br>2 violations: MW>480, MR>130	0 alert
**5**	398.45	89.90	93.81	109.55	3.77	2.56	2.631	Soluble	Yes<br>0 violation	Yes<br>0 violation	0 alert
**9**	488.74	69.92	69.92	63.79	4.57	6.15	4.413	Poorly soluble	Yes<br>1 violation: LogP	No; 4 violations: MW>480, WLOGP>5.6,MR>130, #atoms>70	0 alert
**11**	410.46	106.75	106.75	123.94	3.27	2.75	1.089	Soluble	Yes<br>0 violation	Yes<br>0 violation	0 alert
**12**	534.63	136.21	104.12	104.22	4.17	4.19	4.984	Moderately soluble	Yes<br>1 violation: MW>500	No;<br>2 violations: MW>480, MR>130	0 alert
**13**	359.33	127.69	127.69	149.53	2.87	0.95	0.386	Soluble	Yes<br>0 violation	Yes<br>0 violation	0 alert

***Swiss ADME**;

****Molinspiration**

#**QikPropv6.5**: **vdW Polar SA (PSA)** = 7.0/200.0; **QPlogP for octanol/water** = -2.0/6.5.

TableS7 (B) Pharmacokinetic profile and toxicity of compounds **4**, **5**, **9**, **11-13** predicted by SwissADME, preADMET and Qikprop

entry	GI absorption*	Caco-2**	Caco-2#	BBB permeant*	BBB**	QPlogBB#	MDCK**	MDCK#	Ames test**	hERG inhibition**	HERG K+#
**4**	high	15.856	68	no	0.527	-2.701	0.062	27	Non-mutagen	medium_risk	-6.032
**5**	high	24.452	445	no	0.028	-1.251	0.215	206	Non-mutagen	medium_risk	-4.784
**9**	high	24.188	814	no	5.400	-0.990	0.046	396	Non-mutagen	low_risk	-3.685
**11**	high	16.064	60	no	0.012	-1.493	0.058	76	Mutagen	low_risk	-2.146
**12**	low	23.367	629	no	0.111	-1.198	55.024	679	Mutagen	ambiguous	-8.283
**13**	high	11.320	44	no	0.049	-2.579	1.507	17	mutagen	low_risk	-5.768

*<u>**Swiss ADME**</u>

<u>PreADMET**</u> **Caco-2 cell permeability**: low permeability = less than 4, middle permeability = 4/70; high permeability = more than 70; **BBB penetration:** high absorption to CNS more than 2.0, middle absorption to CNS 2.0/0.1, low absorption to CNS = less than 0.1; **in vitro MDCK cell permeability** (Mandin Darby Canine Kidney) high permeability = more than 500; middle permeability = 25/ 500; low permeability = less than 25.

<u>**QikPropv6.5:**</u> Apparent **Caco-2 Permeability** (nm/sec) = <25 poor, >500 great; **QPlogBB** for brain/blood= -3.0/1.2; Apparent **MDCK Permeability** (nm/sec) = <25 poor, >500 great); **HERG K+** Channel Blockage: log IC_{50} = concern below -5

TableS7 (C) Bioactivity profile of compounds **4**, **5**, **9**, **11-13** predicted by SwissADME, molinspiration and QikPropv6.5.

entry	Target kinase*	mTOR* (probability)§	GPCR Ligand**	Ion channel modulator**	Nuclear receptor ligand**	Enzyme inhibitor**	Kinase inhibitor**	Protease inhibitor**
**4**	yes	yes (0.125)§	Yes	no	yes	yes	no	no
**5**	no	no	no	no	no	yes	no	no
**9**	yes	no	yes	no	yes	yes	no	yes
**11**	no	no	no	no	no	no	no	no
**12**	yes	no	no	no	no	no	no	no
**13**	yes	Yes (0.109)§	no	no	no	no	no	no

<u>***Swiss ADME**</u>

<u>****Molinspiration**</u>

TableS8 Smile strings and CAS number of the selected compounds **4**, **5**, **9**, **11-13**

entry	chemical name/smile	CAS registry number*	ZINC database ID**
**4**	(2R,3R)-2-[4-hydroxy-3-(3-hydroxypropyl)-5-methoxyphenyl]-11-(2-hydroxyethoxy)-2H,3H,4H,5H,6H-phenanthro[2,1-b]pyran-3,8-diol COc1cc([C@H]2Oc3cc(OCCO)c4c(c3C[C@H]2O)CCc2cc(O)ccc2-4)cc(CCCO)c1O	----	ZINC000085568190
**5**	(6aS)-9-[(2S,3R)-3-Hydroxy-2-methyl-1-oxobutyl]-6a-methyl-3-[(1E,3S)-3-methyl-1-penten-1-yl]-6H-furo[2,3-h]-2-benzopyran-6,8(6aH)-dione - Chaetoviridin H CCC(C)C=CC1=CC2=CC(=O)C3(C(=C(C(=O)O3)C(=O)C(C)C(C)O)C2=CO1)C	1308671-12-6	ZINC000072109671
**9**	(3β,4β,7β,9β,10α,23R)-4-(Hydroxymethyl)-7-methoxy-4,9,14-trimethyl-19-norcholesta-5,24-diene-3,23-diol Balsaminol B CC(CC(C=C(C)C)O)C1CCC2(C1(CCC3(C2C(C=C4C3CCC(C4(C)CO)O)OC)C)C)C	1189131-53-0	ZINC000049793204
**11**	1-[1-Oxo-3-(2,3,5-trimethyl-7-oxo-7H-furo[3,2-g][1]benzopyran-6-yl)propyl]-4-piperidinecarboxamide CC1=C(OC2=CC3=C(C=C12)C(=C(C(=O)O3)CCC(=O)N4CCC(CC4)C(=O)N)C)C	859141-12-1	ZINC000002111213
**12**	1-[(3S,3AR,6S,6aR)-3-[[4-(4-phenylphenyl)pyrimidin-2-yl]amino]-2,3,3a,5,6,6a-hexahydrofuro[3,2-b]furan-6-yl]-3-(3-cyanophenyl)thiourea C1C(C2C(O1)C(CO2)NC(=S)NC3=CC=CC(=C3)C#N)NC4=NC=CC(=N4)C5=CC=C(C=C5)C6=CC=CC=C6	1212658-16-6	ZINC000005399642
**13**	N-(3-Hydroxypropyl)-3-[[(4-methyl-2-oxo-2H-1-benzopyran-7-yl)oxy]methyl]-1,2,4-oxadiazole-5-carboxamide CC1=CC(=O)OC2=C1C=CC(=C2)OCC3=NOC(=N3)C(=O)NCCCO	1081140-89-7	ZINC000020728457

* compounds **5**,**9**,**11**,**12**, and **13** are reported in SCI-finder database (https://scifinder-n.cas.org); their corresponding CAS registry numbers as well as several references are available; moreover, the suppliers are indicated in the databases.

** compound **4** is not present in SCI-finder database, no CAS number is registered; the information about compound **4** is available only in ZINC database.

FigureS4 Superimposition of the docking poses of compounds **4**, **5**, **9**, **11-13** with the crystallographic binding conformation of rapamycin.

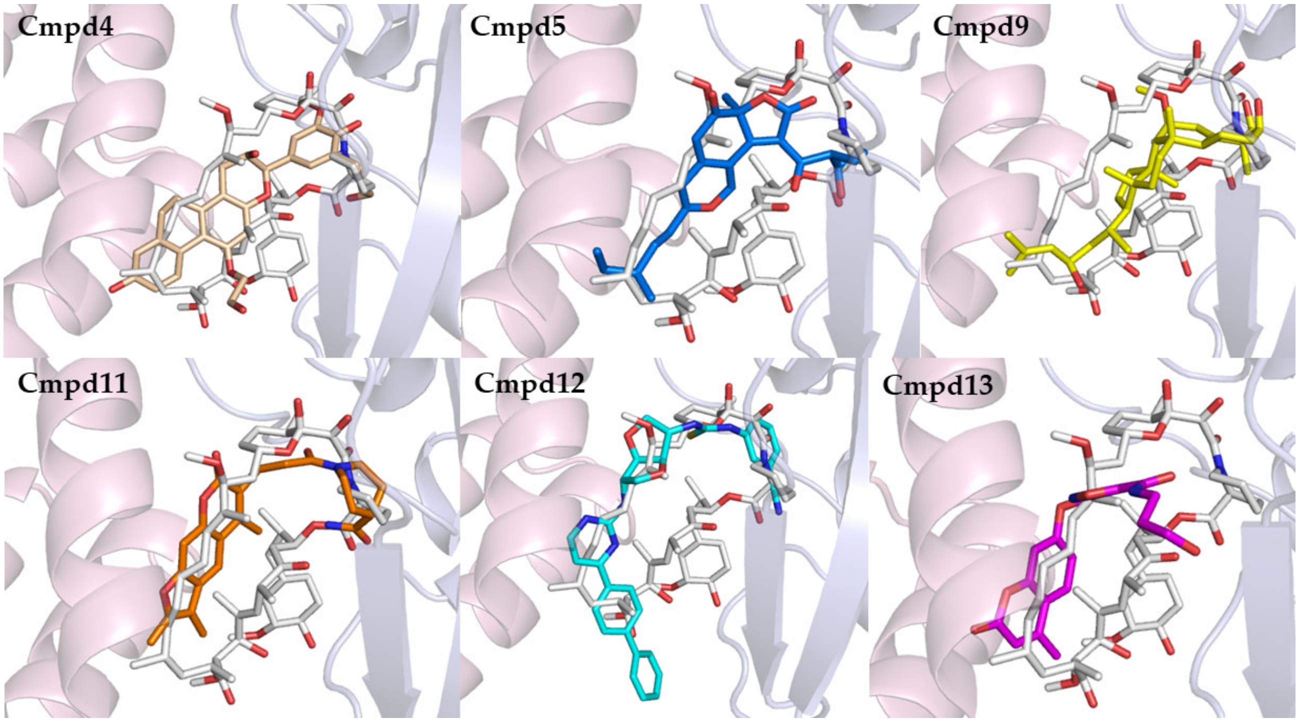

FigureS4. Superimposition of the docking poses of compounds **4** (wheat sticks), **5** (blue sticks), **9** (yellow sticks), **11** (orange sticks), **12** (cyan sticks) and **13** (magenta sticks) with rapamycin (white sticks).

Figure S5

A) Compound 4

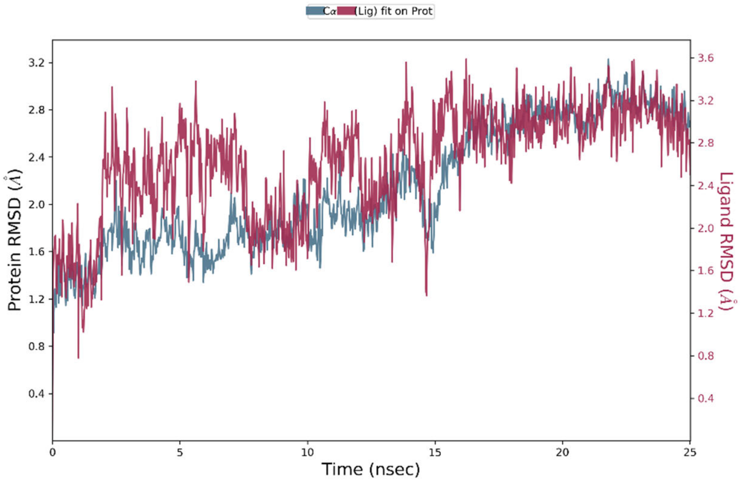

B) Compound 5

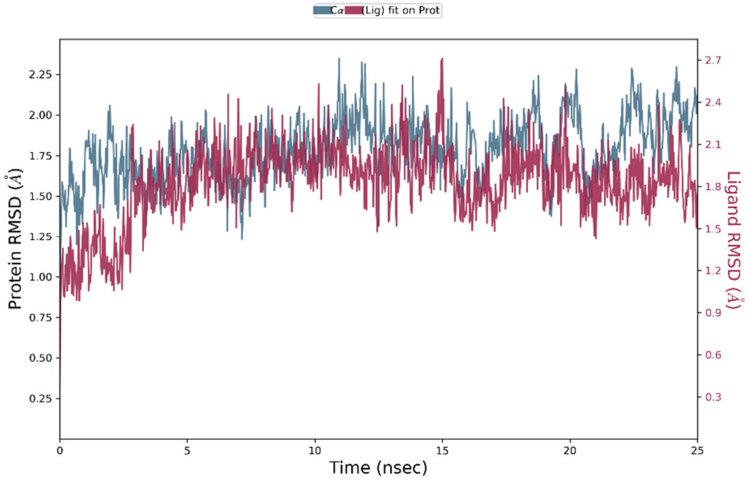

C) Compound 9

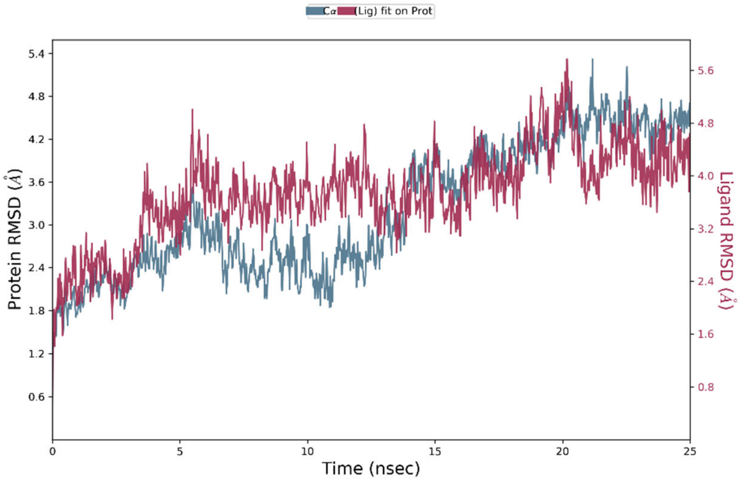

D) Compound 11

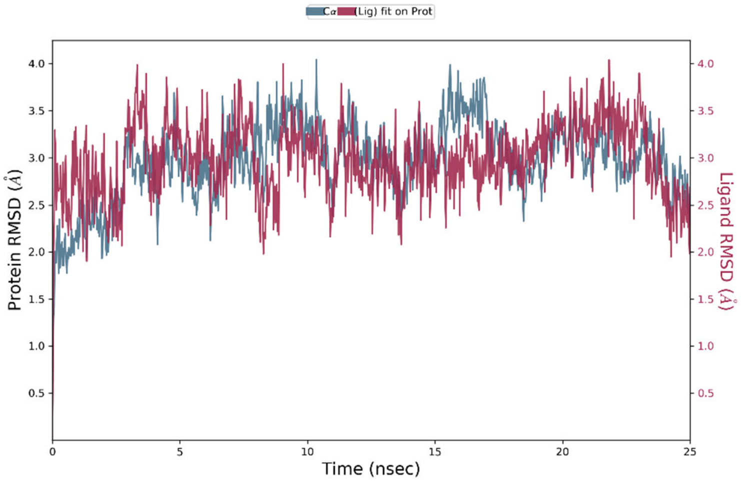

E) Compound 12

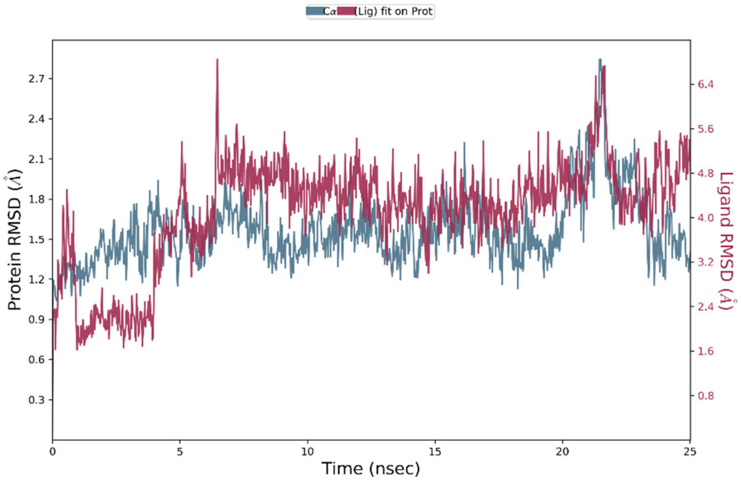

F) Compound 13

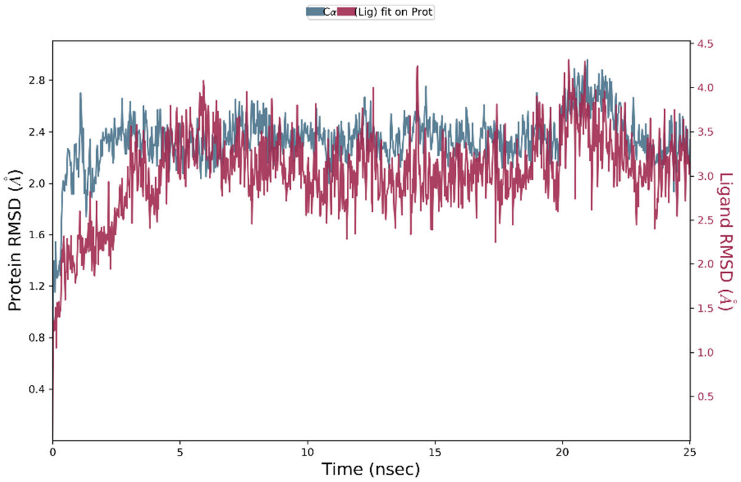

FigureS6. RMSD plots of the ternary complexes obtained from the docking and rescoring procedures.
